# RAG2 mutants alter DSB repair pathway choice *in vivo* and illuminate the nature of ‘alternative NHEJ’

**DOI:** 10.1093/nar/gku295

**Published:** 2014-04-20

**Authors:** Vered Gigi, Susanna Lewis, Olga Shestova, Martina Mijušković, Ludovic Deriano, Wenzhao Meng, Eline T. Luning Prak, David B. Roth

**Affiliations:** 1 Department of Pathology and Laboratory Medicine and Abramson Family Cancer Research Institute, Raymond and Ruth Perelman School of Medicine, University of Pennsylvania, Philadelphia, PA 19104, USA; 2 Departments of Immunology and Genomes & Genetics, Institut Pasteur, CNRS-URA 1961, 75015 Paris, France; 3 Department of Pathology and Laboratory Medicine, Perelman School of Medicine, University of Pennsylvania, Philadelphia, PA 19104, USA

## Abstract

DNA double-stranded breaks (DSBs) can be repaired by several mechanisms, including classical NHEJ (c-NHEJ) and a poorly defined, error-prone process termed alternative NHEJ (a-NHEJ). How cells choose between these alternatives to join physiologic DSBs remains unknown. Here, we show that deletion of RAG2's C-terminus allows a-NHEJ to repair RAG-mediated DSBs in developing lymphocytes from both c-NHEJ-proficient and c-NHEJ-deficient mice, demonstrating that the V(D)J recombinase influences repair pathway choice *in vivo*. Analysis of V(D)J junctions revealed that, contrary to expectation, junctional characteristics alone do not reliably distinguish between a-NHEJ and c-NHEJ. These data suggest that a-NHEJ is not necessarily mutagenic, and may be more prevalent than previously appreciated. Whole genome sequencing of a lymphoma arising in a p53^−/−^ mouse bearing a C-terminal RAG2 truncation reveals evidence of a-NHEJ and also of aberrant recognition of DNA sequences resembling RAG recognition sites.

## INTRODUCTION

Misrepair of double-stranded breaks (DSBs) creates structural genomic lesions (deletions, chromosome translocations, duplications and inversions) that can fuel oncogenic transformation (1,2) . One of the canonical mechanisms responsible for DSB repair, classical non-homologous end joining (c-NHEJ), limits such genomic damage and suppresses tumorigenesis (3,4). By contrast, the loosely defined a-NHEJ pathway is thought to operate with much lower fidelity and has been implicated in oncogenic genome rearrangements, mainly chromosomal translocations, both in cancer and in cultured cells (3–8). Indeed, some human tumors show evidence of upregulated a-NHEJ activity (9–11).

Although a-NHEJ was discovered in cells deficient for c-NHEJ (12–14), a-NHEJ is active even in c-NHEJ-proficient cells in culture (5,15,16) suggesting that mechanisms might exist to limit its usage, presumably to preserve genomic integrity. How cells control choice of a particular pathway (homologous recombination, c-NHEJ or a-NHEJ) for repair of a given DSB has not been determined, and is a question of intense current interest. Most attempts to study pathway choice between c-NHEJ and a-NHEJ to date have employed artificial systems and were carried out in the absence of one or more critical DNA damage/repair factors (6,17–19). This is not ideal for determining the mechanism of pathway choice, as the missing factor might itself be involved. Furthermore, the absence of a key factor (e.g. a component of c-NHEJ) may trigger compensatory changes in repair or damage signaling. Another potential disadvantage is that these approaches may bias the type of junctions produced by a-NHEJ, e.g. disabling end protection mechanisms leading to excessive deletion or production of long single-stranded tails.

V(D)J recombination provides a tractable and physiologically relevant system in which to explore end-joining pathways and regulation of pathway choice. On the face of it, V(D)J recombination has tremendous potential for errors. It introduces DSBs in large numbers of lymphocyte progenitors, and, through end-to-end joining, generates megabase-sized modifications of the genome (20). The system has evolved to minimize aberrant events. For example, the C-terminus of RAG2, while not essential for recombination, is evolutionarily conserved and essential for proper repair (15,21–25). Our understanding of ‘classical’ NHEJ relied heavily on determining requirements for coding and signal joint formation in V(D)J recombination and repair of radiation-induced DNA breaks. Because exposure to a different suite of enzymes and repair ‘platforms’ is likely to affect the fine-structure of the repair junctions, the available comprehensive analyses of V(D)J recombination outcomes collected over the last 30 years provide an ideal basis by which to explore and define end-joining alternatives.

The V(D)J recombinase, comprised of the protein products of recombination activating genes 1 and 2 (the RAG1/2 proteins), cleaves the DNA between an antigen receptor coding segment and a flanking recombination signal sequence (RSS). RSS consist of conserved heptamer and nonamer sequences separated by a spacer of 12 or 23 nucleotides. DSB formation normally requires synapsis of a 12/23 RSS pair, and produces two covalently sealed (hairpin) coding ends and two blunt signal ends (20). Both coding ends and signal ends are then joined exclusively by the c-NHEJ repair machinery that includes the Ku heterodimer (Ku80 and Ku70), DNAPKcs, XRCC4/DNA ligase IV, Artemis and XLF (20).

Our previous work suggests that it might be possible to alter the end-joining environment encountered by broken DNA ends by mutating RAG1/2 without perturbing the end-joining factors themselves (15,22). Indeed, a particular C-terminally truncated RAG2 mutant termed FS361, identified in our lab, allows coding ends to abnormally access a-NHEJ ((15), Supplementary Figure S1A). This was assessed using RAG expression vectors transfected into fibroblasts along with an extrachromosomal substrate specifically designed to detect joints bearing both excessive deletion and microhomologies that have been considered characteristic of such repair (6,15,26,27). This implies that RAG2's C-terminus is important for control of pathway choice, at least in this artificial system.

A focus on RAG2 is additionally supported by studies in which the consequences of germline mutations in the RAG2 C-terminus have been examined in whole mice (23–25). Though not addressed directly in those reports, the recombinant V(D)J junctions that were observed raised the possibility that each of the C-terminal mutations may have had an impact upon pathway choice. Hence, to seek a definitive evidence of functional alternative pathways, specifically a-NHEJ, without changing components of c-NHEJ, we generated homozygous knock-in mice bearing the FS361 mutation (RAG2^FS/FS^) that exhibited the highest a-NHEJ signal among those tested in transfection studies. In addition to establishing physiological relevance of pathway choice *in vivo*, this minimally manipulated, physiologically relevant system is ideally suited to investigate the hypothesis that a-NHEJ is error-prone and makes a disproportionate contribution to the oncogenic genome rearrangement.

We find that repair pathway choice is enforced during V(D)J recombination *in vivo*, in both c-NHEJ-proficient and c-NHEJ-deficient mice, and that RAG2's C-terminus is critical for this regulation. Surprisingly, we find that the way in which a-NHEJ handles broken DNA ends is strongly context-dependent: signal joints show reported features of a-NHEJ, whereas coding joints do not. In fact, joining of RAG-generated coding ends by a-NHEJ in our system produced junctions that were largely indistinguishable from those arising from c-NHEJ. Whole genome sequencing of a lymphoma derived from a RAG2^FS/FS^;p53^−/−^ mouse showed that the majority of genomic lesions similarly lack distinctive features of a-NHEJ. However, some lesions, including those in known oncogenes, appear to arise from ectopic recombination between DNA sequences fortuitously resembling RSS (cryptic RSS, cRSS). Together these data show that RAG2's C-terminus alters accessibility to a-NHEJ and preserves genomic stability by reducing inappropriate recognition of cRSSs.

## MATERIALS AND METHODS

### Mice

We obtained wild-type (WT) (Taconic), Ku80 knock-out (KO) (The Jackson Laboratory, (28)), RAG2 KO (The Jackson Laboratory) and RAG2^C/C^ that we renamed RAG2^del352/del352^ (29). RAG2^FS/FS^ mice were generated by Ingenious Targeting Laboratories as described in Supplementary Figure S1. The nucleotide sequence of the entire RAG2 ORF was verified by sequencing genomic DNA from somatic tissues of the knock-in mouse. Rag2^FS/FS^ and RAG2^del352/del352^ were bred with Ku80 KO mice to generate doubly deficient mice. Genotyping of all mice was performed by polymerase chain reaction (PCR) of tail DNA as described ((28,29), Supplementary Figure S1). The animals’ care was approved by UPenn Institutional Animal Care and Use Committee (IACUC) Protocol no. 803893.

### Flow cytometry

Cells from thymus, bone marrow (BM) and spleen were obtained from the indicated genotypes and stained for B cell (B220, CD43, IgM) and T cell (CD4, CD8, thy1.2, TCRβ, CD25, CD44) markers. Fluorescence-activated cell sorting (FACS) analysis was done using the BD LSR II and FlowJo software.

### PCR for CJs, SJs and interchromosomal rearrangements

Genomic DNA from thymus and BM were prepared from 6- to 9-week-old mice for WT RAG2^FS/FS^ and RAG2^del352/del352^ characterization and 4-week-old mice for joints from Ku80 deficient backgrounds. Genomic DNA for coding joints (CJ, 200 ng) or signal joints (SJ)/interchromosomal (500 ng) were amplified by PCR. CJ/SJ primers were described previously (25) aside from Vb10 SJ, Vh7183 CJ that are described below. For interchromosomal rearrangements we used nested PCR with primers described below. All PCR products were cloned using the TA cloning kit. For SJ, single clones were subjected to PCR with TOPO TA primers and products were then digested with ApaL1 to detect precise joints. Plasmid DNA from resistant SJs clones, all CJs and interchromosomal rearrangement clones were prepared and Sanger sequenced.

Interchromosomal rearrangement primers 5′-3′:

Dd2 F1: CAAGCATTAGACAGTAAGTACCCAG

Dd2 F2: GCCAACCACTTTGATAGTCTGTGGCTTG

Db1 R1: GAGTAATCGCTTTGTGTGCATCACA

Db1 R2: CATTCTGGATCTAAACACATCTAGGCTTGC

Vb10 SJ: CTCAGTGAGACTCATCGGTGC

Db1.1 SJ: CATTAGCTCGCATCTTACCAC

VhE: GTGGAGTCTGGGGGAGGCTTA

Vh7183: CCAAGAAGACCCTGTACCTGCAAATGA

Jhintronic: CTCCACCAGACCTCTCTAGACAGC

Jh4: TCAAATGAGCCTTCCAAAGTCC

### Spectral karyotyping

For metaphase preparations, primary tumor cells were grown in Roswell Park Memorial Institute media (RPMI) supplemented with 20% fetal bovine serum, l-glutamine and β-mercaptoethanol for 2 h and exposed to colcemid (0.025 μg/ml) and BrdU (28 μg/ml) for an additional 2 h at 37°C. Then, cells were incubated in 40 mM KCl for 25 min at 37°C, fixed in fixative solution (75% methanol/25% acetic acid) and washed twice in the fixative. Cell suspensions were dropped onto pre-chilled glass slides and air-dried. Spectral karyotyping (SKY) was performed using the Applied Spectral Imaging mouse SKY probe according to the manufacturer's instructions to determine chromosomal rearrangements. The slides were analyzed using a Nikon Eclipse 80i microscope. SKY images were captured and karyotyped using an Applied Spectral Imaging system.

### Adjacent direct repeat quantification

Adjacent direct repeats (ADRs) were scored in a fashion such that repeats, which were generated randomly, were removed. First, we calculated the insertion probabilities of A, T, G, C nucleotides. For that purpose, we omitted any insertions that could have been generated as P nucleotides and calculated nucleotides probabilities for each TCRβ locus separately. Second, we calculated the probability of a certain ADR to arise by multiplying the relevant nucleotide probability times the number of total junctions (e.g. *P*_(n)_ × *P*_(n)_ × *P*_(n)_ × *N*) for each locus. Any multiplication above 1 meant that, by chance, there is a likelihood of one junction to have a certain ADR. Therefore, to be stringent, we scored as statistically significant multiplication <0.5.

### Whole genome sequencing

Detailed procedures can be found in (30). Briefly, genomic DNA from Rag2^FS/FS^;p53^−/−^ tumor and liver (control) tissues was purified. Paired-end libraries were generated according to manufacturer's recommendations. Libraries were then analyzed for size distribution and sequenced on an Illumina HiSeq 2000. Sequence coverage was calculated by (no. of reads adjusted to duplication × average inset size in bp)/bp in the mouse genome). We obtained 35- and 37-fold coverage for tumor and liver, respectively. Filtering pipeline is described in (30). Potential genomic candidates were validated by PCR using custom designed primers against tail DNA. Lesions that were validated as tumor specific were cloned (TOPO TA) and Sanger sequenced.

cRSS definition: To score for aberrant rearrangements involving cRSSs, we used the reported Notch1 cRSSs as a guideline (31). We calculated the sum of nucleotides matches in a 12- and a 23-RSS to a consensus one. We chose this approach in order to adhere to the 12/23 rule (20). Notch1 cRSSs have 19 nucleotide matches to a consensus RSS (31), hence, any paired cRSSs in our cohort that scored 19 or above were counted as positive.

### Notch1 and Bcl11b intragenic deletions

Genomic DNA from thymocytes was prepared using Roche High Pure PCR template preparation kit according to manufacture recommendations. Nested PCR was preformed to detect intergenic deletion for both genes. PCR protocols and primers were described previously (31,32). Modified primers for Bcl11b are described below. For a negative control, we used genomic DNA from R2 KO liver. PCR samples were analyzed by electrophoresis on agarose gels; identification of a band at the appropriate size was considered as an indication of rearrangement. Four samples from RAG2^FS/FS^ mice and positive RAG2 WT were purified, TOPO TA cloned, and Sanger sequenced.

Primers:

Bcl11b F1: CTCTCCAATCCTGTGGTCTCTTAC

Bcl11b F2: GGGAACGCTTTTCGGCCTTACTTG

Bcl11b R1: GTCAGCCTAAGGCTACAGCACATTATG

Bcl11b R2: CTCTTCTGCACAGCTTTCCCTCTG

### Statistical analysis

A two-tailed unpaired *t*-test was applied for extrachromosomal recombination efficiency and thymus cellularity. For all other statistical analysis, we used the Chi-squared test of independence.

## RESULTS

### Signal joints from RAG2^FS/FS^ mice show reported features of a-NHEJ

To explore pathway choice control at the chromosomal level in repair-proficient animals, we generated FS361RAG2 knock-in mice (Supplementary Figure S1B–D). We chose this mutant because it yields the highest levels of a-NHEJ of any RAG mutant tested *in vitro* (Supplementary Figure S1A and (15,22)). Specifically, this allele yields 2.5- to 5-fold higher levels of a-NHEJ than ‘core’ RAG2 (truncated at amino acid 383; (33,34)) (Supplementary Figure S1A, (15)). RAG2^FS/FS^ knock-in mice exhibit a mild block in early lymphocyte differentiation (Supplementary Figure S2) closely resembling that described for core RAG2 mice (RAG2^C/C^), and, as expected, remain capable of generating mature lymphocytes (29,35).

Initially, we examined signal joints that are informative for two reasons. First, the sequence of these junctions is noncoding, and thus not subjected to selective pressures during lymphocyte differentiation. Second, signal joints have a well-defined structure, being formed by blunt ligation of the signal ends. In a signal joint, the two RSSs abut and although the occasional insertion of nucleotides is seen, deletions in either RSS is rare. Hence, nucleotide sequence features considered characteristic of a-NHEJ (excessive deletions, long insertions and junctional microhomologies) are readily identified.

Signal joints arising from recombination at the T cell receptor (TCR) β locus in RAG2^FS/FS^ mice exhibited a significant increase in imprecise joints (63/140, 45%) compared with age-matched WT controls (34/159, 21.4%) (*P* < 0.0001, Table 1). RAG2^FS/FS^ junction sequences showed a significant increase in deletions (32/63, 50.7%, versus 6/34, 17.6%, seen in junctions from WT mice, *P* < 0.001; Table 1). Strikingly, at least 50% of deleted signal ends from RAG2^FS/FS^ mice had deletions greater than five nucleotides, a feature not observed in over 150 signal joints from WT mice (Table 1, Supplementary Figure S3). Other sequence features considered characteristic of a-NHEJ not observed in junctions from WT mice included occasional microhomologies (5/140 junctions, ranging from 2 to 9 bp), and large insertions (350 and 26 bp; Supplementary Figure S3). A similar trend was previously observed in mice bearing the less severely truncated core RAG2 allele (25) and also the RAG2 T490A allele (in which the protein degradation signal is ablated; (23)) signifying the importance of an intact RAG2 C-terminus. In aggregate, these features indicate that signal ends are abnormally available to a-NHEJ in RAG2^FS/FS^ mice, suggesting that repair pathway choice is disabled.

**Table 1.** RAG2^FS/FS^ mice exhibit a-NHEJ at the signal joints

	Vβ14-Dβ1 Inv	Vδ5-dδ2 Inv	Vβ8.3-Dβ1.1 Del	Vβ10-Dβ1.1 Del
**WT**				
Precise joints	68/79 (86%)	19/28 (68%)	19/27 (70%)	19/25 (76%)
Imprecise joints	11/79 (14%)	9/28 (31%)	8/27 (30%)	6/25 (24%)
*N* addition	10/11 (91%)	9/9 (100%)	8/8 (100%)	6/6 (100%)
Deletion	0/11 (0%)	3/9 (30%)	3/8 (37.5%)	0/8 (0%)
%Deletions >5 bp	0%	0%	0%	0%
Miscleavage	1/11 (9%)	0/9 (0%)	0/8 (0%)	0/6 (0%)
Microhomology ≥2 bp	0/11 (0%)	0/9 (0%)	0/8 (0%)	0/6 (0%)

**RAG2^FS/FS^**				
Precise joints	47/70 (67%)	6/26 (22%)	15/28 (54%)	9/16 (56%)
Imprecise joints	23/70 (33%)^a^	20/26 (78%)^a^	13/28 (46%)	7/16 (44%)
*N* addition	16/23 (69%)^b^	14/20 (70%)	13/13 (100%)	6/7 (86%)
Deletion	9/23 (39%)	17/20 (85%)	4/13 (30%)	2/7 (29%)
%Deletions >5 bp	10/14 (71%)	16/30 (53%)	4/4 (100%)	0%
Miscleavage	3/23 (13%)	4/20 (20%)^c^	5/13 (38%)	2/7 (29%)
Microhomology ≥2 bp	2/23 (8.5%)	2/20 (10%)	0/13 (0%)	1/7 (14%)

Sequences analyses from two to three independent mice were combined. Frequencies of N nt additions, deletions, miscleavage and microhomology were calculated out of imprecise junctions. Deletions >5 bp is calculated out of total deleted ends (5 bp deletion was the longest seen in WT and thus chosen as a cutoff). Miscleavage represents coding end sequences at the ends.

^a^*P* < 0.01 versus WT.

^b^One insertion was 350 bp from Vb3 region (Supplementary Figure S3).

^c^Two junctions underwent open-shut intermediate (indicated in blue, Supplementary Figure S3).

### Coding joints from RAG2^FS/FS^ mice fail to exhibit reported features of a-NHEJ

We next examined coding joints formed at immunoglobulin (Ig) and TCR loci. Unlike signal joints, nucleotide sequences of coding joints from the two models (RAG2^FS/FS^, *n* = 136; WT, *n* = 94) were qualitatively similar (Table 2, Supplementary Figure S4). Because of the potential for bias imposed by biological selection for productive rearrangements (36), we also analyzed coding joints from (noncoding) D–J rearrangements and from sorted CD4/CD8 double negative thymocytes (which are not subject to selection for productive rearrangements; Table 2, data not shown). Again, there was no qualitative difference between RAG2^FS/FS^ and WT mice. Finally, we looked at the third complementarity-determining region (CDR3) sequence of antibody heavy chain gene rearrangements in genomic DNA of splenocytes from WT and RAG2^FS/FS^ mice. The CDR3 is generated by V(D)J rearrangement and is influenced by nontemplated additions and deletions. Significant shifts in CDR3 length, therefore, can serve as indirect evidence of a-NHEJ repair. CDR3 spectratyping of VH606 and VH558 rearrangements to JH2 from splenocytes revealed no significant differences (Supplementary Figure S4F).

**Table 2.** No detectable a-NHEJ repair at antigen receptor coding joints in RAG2^FS/FS^ mice

	Vβ6/7/8-Jβ2	Vβ10-Jβ2.1	Vβ14-Jβ1.1	Dβ2-Jβ2.6	Vh7183-Jh4
**WT**	*n* = 23	*n* = 16	*n* = 11	*n* = 15	*n* = 17
Deletion in V region	80%/−3.6	100%/−4.2	36%/−3.5	69%/−2.2	82%/−2.92
4 bp deletion	31%	37.5%	25%	20%	21%
Deletion in J region	95%/−4.4	94%/−3	63%/−5.4	87%/−5.38	94%/−5.93
4 bp deletion	41%	27%	57%	46%	57%

**RAG2^FS/FS^**	*n* = 31	*n* = 32	*n* = 14	*n* = 17	*n* = 21
Deletion in V region	77.5%/−4.63	97%/−4.45	43%/−3.3	53%/−2.9	48%/−2.7
4 bp deletion	38%	48%	33%	22%	20%
Deletion in J region	90%/−5.29	81%/−3.1	64%/−5	82%/−4.78	76%/−6.12
4 bp deletion	57%	23%	56%	43%	56%

Sequence data from two to three independent thymic or BM genomic DNA samples were combined. The frequency of deleted ends at the V or J regions was from total events. Average of base pair (bp) deletion and frequency of >4 bp deletion was calculated out of deleted events only (4 bp was the average deleted length in WT junctions and thus chosen as a cutoff).

We considered three reasons for the lack of distinctive sequence features at coding joints formed in RAG2^FS/FS^ mice. (i) The RAG2 FS allele might selectively enforce pathway choice for signal ends, but not for coding ends. (ii) Competition from c-NHEJ could render the ‘background’ of normal coding joints too high to allow us to detect rare joints formed by a-NHEJ. (iii) Coding joints formed by a-NHEJ may not be structurally distinctive. To explore these possibilities further, we used more sensitive assays to detect coding joints formed by a-NHEJ.

### RAG2^FS/FS^ mice show increased inter-chromosomal rearrangements between antigen receptors together with excessive deletions

a-NHEJ has been firmly implicated in chromosome translocations in various end joining-deficient backgrounds, and the translocation junctions show characteristic sequence features such as microhomologies and excessive deletions (3,5,18). We therefore investigated our RAG2^FS/FS^ mice for elevated levels of translocations. In particular, abnormal translocations between the TCRβ and TCRδ loci, located on chromosomes 6 and 14, respectively, has previously been observed in mice bearing another RAG2 mutation (24). The D regions involved are flanked by a 12- and a 23-RSS that can lead to signal joints or coding joints products in a translocation junction (Supplementary Figure S5A). We detected translocations in four out of five RAG2^FS/FS^ mice, but not in thymocytes from WT controls (*n* = 4, *P* < 0.02). We observed excessive deletions in 6/10 junctions with evidence in one case for a 2-bp junctional microhomology (Supplementary Figure S5B). Unexpectedly, none of the junctions retained the D region coding end sequences. Because of this we could not establish whether the extensively deleted translocation junctions were abnormal coding joints or signal joints. Nonetheless, the observation of chromosomal translocations suggests that pathway choice is defective in the RAG2 mutant mice, in agreement with our analysis of signal joints.

### Coding joint formation bypasses Ku80 deficiency in RAG2^FS/FS^ mice

Ku80^−/−^ mice lack a critical component of c-NHEJ, and are thus severely deficient for both coding and signal joints, leading to a complete block in lymphocyte differentiation at the proB/proT stage (28). We reasoned that if pathway choice were no longer imposed by C-terminally truncated RAG2, the joining of V(D)J recombination intermediates, no longer restricted to c-NHEJ only, might become possible. In other words, lack of joining in Ku80-null animals might be rescued by an alternative pathway in Ku80/RAG2FS double mutants. Furthermore, nucleotide sequence analysis of the V(D)J junctions formed in these mice (in the absence of c-NHEJ) should help interpreting the coding joints formed in RAG2^FS/FS^ mice.

**Figure 1.**
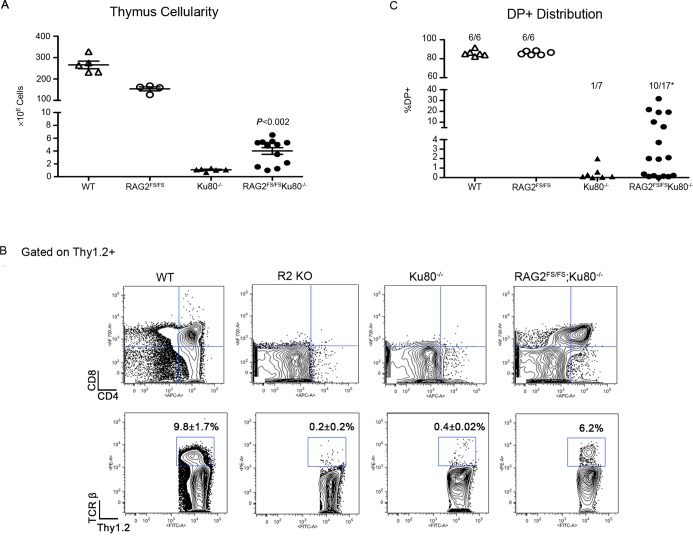
RAG2^FS/FS^ mutant can bypass Ku80 deficiency. Mice from the indicated genotypes were sacrificed at 4 weeks for analysis. (**A**) Thymus cellularity by trypan blue. *P* versus Ku80^−/−^. (**B**) Thymocytes were stained with antiThy1.2 + FITC, antiCD4-APC, antiCD8-AF700 and antiTCRβ-PE. Double positive (DP) populations and TCRβ were calculated by gating on Thy1.2+ cells. (**C**) Distribution of DP cells; only DP >1% was considered as positive. Number of mice analyzed is indicated. *P < 0.05 versus Ku80^−/−^.

RAG2^FS/FS^;Ku80^−/−^ double mutants demonstrated a significant (∼4-fold, *P* < 0.05) increase in thymus cellularity compared with Ku80^−/−^ mice, suggesting a partial bypass of the developmental block (Figure 1A). FACS analysis showed that T cells progressed into the CD4/CD8 double positive (DP) stage in over half of the animals (10/17, *P* < 0.05, Figure 1B–C). As expected, Ku80^−/−^ mice lacked CD4/CD8 positive cells except for one mouse that had a very small DP population (Figure 1C, (28)). Because developmental progression is linked to successful V(D)J recombination, these indications invited further examination of the possibility that the RAG2FS allele rescued TCRβ rearrangement.

**Figure 2.**
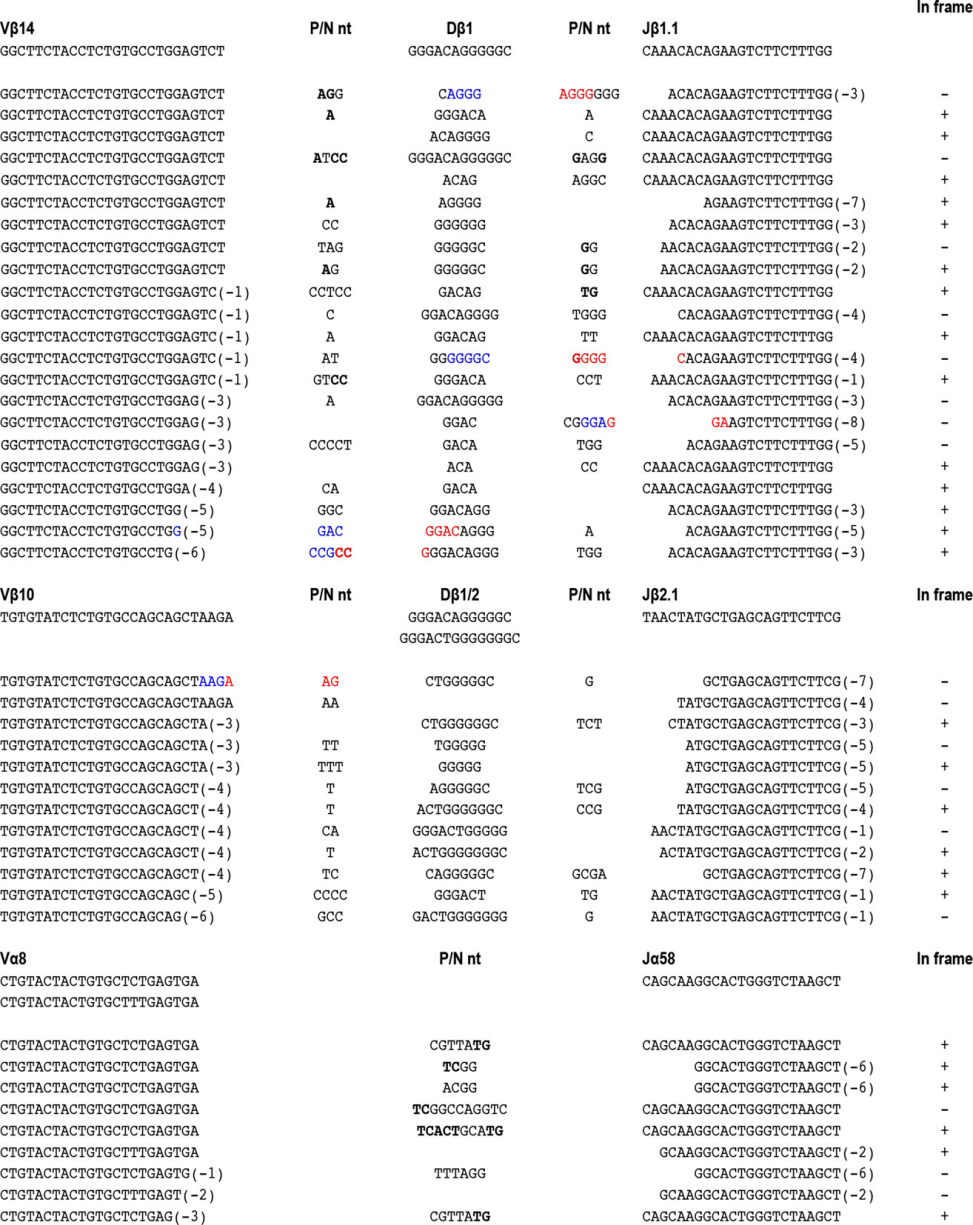
Sequence analysis of TCRβ and TCRα junctions from RAG2^FS/FS^ Ku80^−/−^ mice. Genomic DNA was prepared from RAG2^FS/FS^;Ku80^−/−^ thymocytes (*n* = 2–4). TCR Vβ14-Jβ1.1, TCR Vβ10-Jβ2.1 and TCR Vα8-Jα58. Germline sequences are indicated at the top. Capital letters in the middle of the junction indicate N nt, capital bold letters represent P nt. Deletions are in parentheses and blue/red represents the adjacent direct repeats (ADRs). +/− indicates in/out of frame rearrangements, respectively.

We detected cell surface TCRβ expression in one double mutant mouse (Figure 1B), and demonstrated V(D)J joining via PCR analyses in several other RAG2^FS/FS^;Ku80^−/−^animals. We detected no TCR rearrangements in Ku80^−/−^ mice, in accordance with previous work (28). In the double mutants, we observed TCRβ and TCRα rearrangements (Vβ14, Vβ10 and Vα8) (Figure 2). DNA sequence analysis revealed complete (V to D to J) and diverse coding joints. The FS allele can, therefore, substantially rescue joining of coding ends and bypass the joining defect that is otherwise observed when Ku80 is not present. The joints we observed were formed in the absence of c-NHEJ, and are therefore generated, by definition, by a-NHEJ. We conclude that the C-terminal RAG2 truncation ablates pathway choice control, allowing coding ends to be joined via a-NHEJ.

### Unusual characteristics of coding joints in RAG2FS/Ku80 double mutants

The rescued, Ku80-independent, coding joints lacked features of a-NHEJ that have been reported in other systems (7,37). As in the RAG2^FS/FS^ mice, we saw neither a dependence upon microhomology nor unusually large deletions (Figure 2). These data suggest that, in the context of coding joint formation, a-NHEJ does not display attributes commonly observed in other systems. We did, however, observe an unusual feature: short, three to five nucleotide repeats (termed: Adjacent Direct Repeats (ADRs)) were evident in many of the junctions formed at the Vβ14 locus (Figure 2) in double mutant mice. By a conservative analysis designed to minimize counting repeats generated by chance (see ‘Materials and Methods’ section), 5/22 (23%) Vβ14 junctions contained ADRs. The sequence features of ADRs (being in direct orientation and immediately adjacent) imply a mechanism in which processed ends with complementary extensions (acquired through addition of P or N nucleotides) are annealed and then displaced by a gap-filling polymerase before ligation (Supplementary Figure S6A). Efficient strand displacement is not a known characteristic of either polλ or polμ (38,39) which, along with TdT, are the polymerases thought to be associated with c-NHEJ (40). Pol β, another member of the polX polymerase family, has gap-filling and strong strand-displacement properties (38) and while active in base excision repair (41), has not been described as a factor in c-NHEJ.

The high prevalence of ADRs at the Vβ14 locus prompted us to re-examine coding joints from this locus in WT RAG2 and RAG2^FS/FS^ mice. We found that 1/11 TCR rearrangements from WT RAG2 and 1/14 from RAG^FS/FS^ mice exhibit this feature (Supplementary Figure S4C). We also detected similar levels of ADRs upon review of published coding joint sequences at Vβ14 collected from WT mice (3/47 and 3/40; (23,25), respectively, Supplementary Figure S6B). Though initially observed at Vβ14, ADRs can also be detected elsewhere (Vβ10, Vβ6–8, Dβ2; Supplementary Figure S6B). The frequency of ADRs is increased in the absence of Ku80, implying an association with a-NHEJ. However, like other reported a-NHEJ sequence features (6), they are also observed in junctions from c-NHEJ-proficient animals.

Together, these data allow us to draw the following conclusions. Ends that are formed in the absence of RAG2's C-terminus in c-NHEJ-proficient mice are accessible to a-NHEJ, as shown by signal joints bearing features characteristic of a-NHEJ, and also by the coding joints that are rescued in Ku80-deficient mice. Thus, the C-terminus of RAG2 is important for restricting the end-joining pathways that can repair RAG-generated DSBs *in vivo*. Additionally, our results suggest that Ku80-independent repair is not a disordered, unregulated alternative to c-NHEJ, because rescued coding joints exhibit uniform structural features, resembling junctions formed in RAG2 WT and RAG2^FS/FS^ mice.

### A more extensive RAG2 C-terminal truncation also generates aberrant V(D)J joints and rescues coding joint formation in Ku80-deficient mice

In the FS allele, the normal RAG2 sequence truncates at amino acid 361, but is followed by a novel stretch of 28 amino acids at its C-terminus before the protein sequence terminates at a fortuitous stop codon (15). Through whole genome sequencing, we discovered that the core RAG2 allele in the RAG2^C/C^ mice (derived in the laboratory of M. Schlissel) is not as reported (29) and is a more extensive truncation that terminates at amino acid 352 (instead of the reported 383), with five extra C-terminal amino acids encoded by the targeting vector (Supplementary Figure S7A). We used mice homozygous for this allele, which we rename RAG2del352, to verify the results obtained with the FS allele.

We analyzed signal joints and interchromosomal rearrangements from homozygous RAG2^del352/del352^ mice and TCR rearrangements from RAG2^del352/del352^; Ku80^−/−^ homozygotes and found results similar to those obtained with RAG2 FS allele (Supplementary Figure S7B–D). These data extend the results obtained with RAG2^FS/FS^ mice, indicating that the phenotype is not an artifact of the C-terminal extension encoded by the FS allele, and underscore the importance of the RAG2 C-terminus in repair pathway choice *in vivo*.

### a-NHEJ in aberrant genomic rearrangements from a RAG2^FS/FS^;p53^−/−^ lymphoma

**Figure 3.**
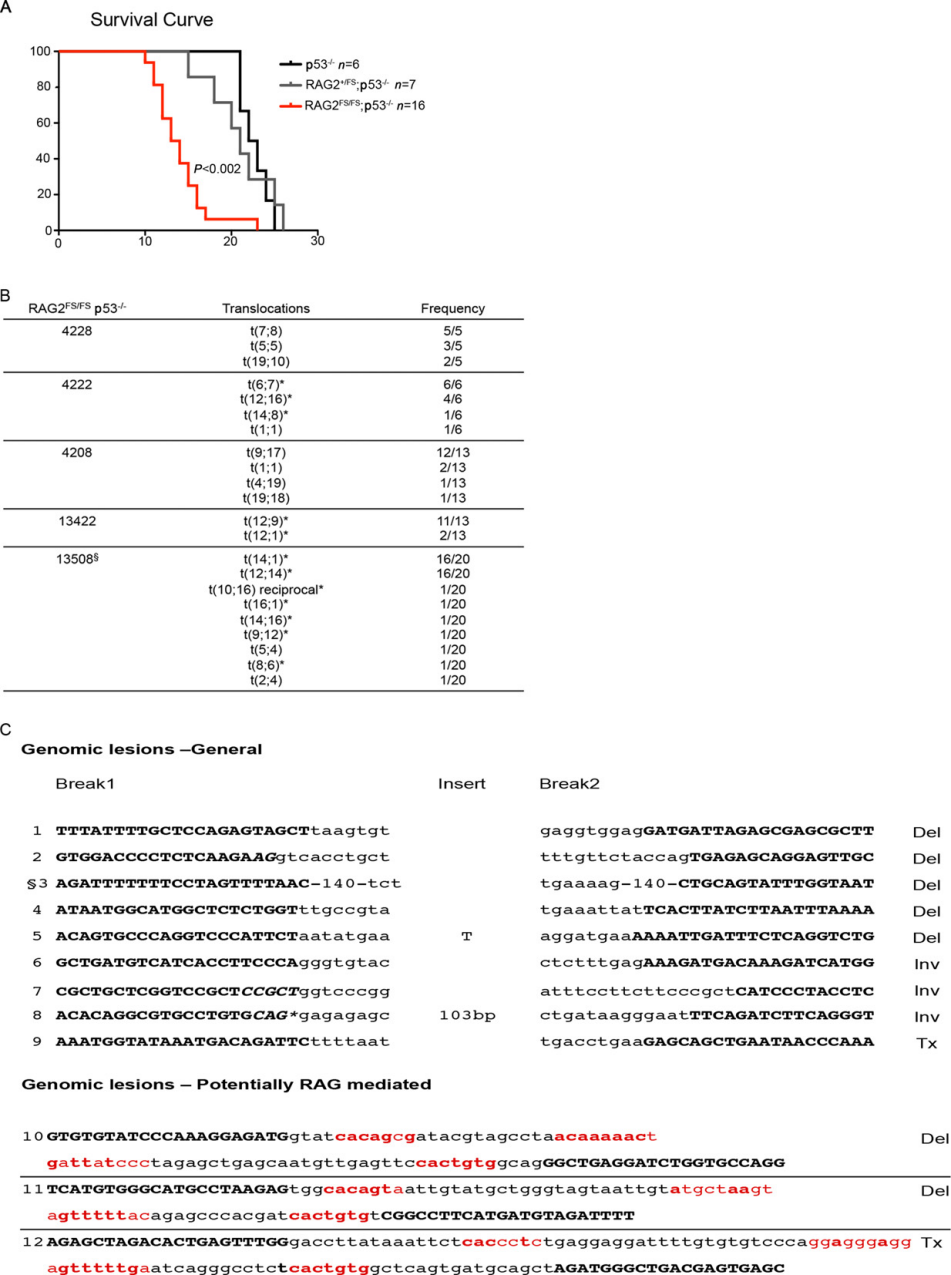
RAG2^FS/FS^;p53^−/−^ mice develop rapid lymphoma with genomic instability. (**A**) Kaplan–Meier survival curve of the indicated genotyped. *P* versus p53^−/−^ mice. (**B**) SKY analysis of RAG2^FS/FS^;p53^−/−^ T cell lymphomas. * indicates antigen receptor chromosome. (**C**) Genomic lesions from RAG2^FS/FS^;p53^−/−^ tumor 13422. The lesions are divided into two groups: General and potential V(D)J mediated. Bold capital letters represent the junction sequence; small letters represent the reference genome at the break points, bold italic letters are microhomologies, red small letters are the cRSSs with bold red representing matches to consensus RSS. Configuration of lesions are indicated to the right; Del, deletion; Inv, inversion; Tx, translocation. ^§^140 bp homology at the junction. *cagcaccacaaacaggacctagtcccgcactgcttggcgataactgcctagctacagggctaggtgctctttggatgcacatgcgcttcactgcacaaaatca.

Several laboratories have reported a connection between a-NHEJ, chromosomal translocations, and accelerated lymphomagenesis (3–5,18). Indeed, c-NHEJ KO mice crossed to a p53-deficient background develop lymphomas with chromosome translocations mediated by a-NHEJ (3,4). To test the possibility that structural features reported for a-NHEJ might exist in genomic lesions from lymphomas, we generated RAG2^FS/FS^;p53^−/−^ mice. Thymic lymphomas emerged rapidly, with median survival of 13.5 weeks (versus 22.5 weeks in p53^−/−^ mice) (*P* < 0.002, Figure 3A). Tumor cells expressed surface CD4 and CD8 with variable amounts of surface TCRβ, implying that these lymphomas originated from immature thymocytes (Supplementary Figure S8A). SKY analysis of these thymomas showed a wide spectrum of chromosome translocations, including but not limited to chromosomes bearing antigen receptor loci (Figure 3B).

To determine whether sequence features of a-NHEJ might be apparent in these genomic lesions, we employed our previously established analysis pipeline to detect structural variants (translocations, deletions, inversions and duplications) in paired-end whole genome sequence data (30). We identified 19 genomic rearrangements in tumor 13422, including translocations and intrachromosomal rearrangements (deletions, inversions, Figure 3C). Seven rearrangements were the result of normal antigen receptor junctions (Supplementary Figure S8B). The remaining 12 rearrangements were aberrant junctions: 7 deletions, 3 inversions, and 2 translocations. Both of the detected translocations were between chromosome pairs previously identified as having undergone an exchange by SKY analysis of this same tumor (Figure 3B).

Three aberrant junctions showed large insertions or microhomologies (Figure 3C). One junction had both a 3 bp microhomology and a large insertion (103 bp) comprised of a duplicated sequence centromeric to the break point and five nontemplated nucleotides, a structure highly suggestive of a-NHEJ. The other two junctions had 2 and 5 nt microhomologies. A third feature associated with a-NHEJ, excessive deletion, can be measured only if an initial break site is known, which is rarely the case for random rearrangement. However, the immature T cell phenotype of these tumors (Supplementary Figure S8A), together with evidence of V(D)J recombination in our example suggested it might be worthwhile to examine junctions for evidence of having been RAG-generated. In such cases, we could assume cleavage at sequences fortuitously resembling RSSs (cRSS), and take advantage of this to score deletion. Indeed, we were able to identify three junctions associated with credible cRSS bearing identifiable heptamer and nonamer sequences at both ends (Figure 3C). Two of the junctions exhibited a typically limited amount of deletion from the cRSSs, but one exhibited more extreme deletion from each breakpoint (15 bp on one side and 16 bp on the other), consistent with a-NHEJ. This junction was created by a translocation. In summary, only one-third of the 12 aberrant rearrangements (two inversions, one deletion and one translocation) exhibited characteristics reported for a-NHEJ. However, the remaining junctions cannot be unequivocally assigned to either a-NHEJ or c-NHEJ because our findings show that a-NHEJ junctions are not necessarily distinct from those formed by c-NHEJ.

### RAG2^FS/FS^ mice show increased cryptic RSS usage

Illegitimate V(D)J recombination in which a *bona fide* RSS is joined to a cRSS or two cRSSs are joined together has been well documented in human lymphomas and in mouse lymphoma models (31,32,42,43). SKY analysis of tumors from RAG2^FS/FS^;p53^−/−^ mice showed a high percentage of the translocations occurring at chromosomes devoid of antigen receptor loci (66% of breaks, Figure 3B). This observation suggests that these translocations may have arisen through recognition of cRSSs. This is supported by our sequencing analysis described above. Moreover, our whole genome sequencing identified two deletions involving potential cRSSs that have been implicated in oncogenesis (*Trmt2a* (44), *TNFR2* (45) and *CD30* (46); Figure 3C junctions 10 and 11). Hence, we wanted to investigate whether the FS allele alone (in the presence of p53) increases use of cRSS. We employed PCR to assay for intragenic deletions in two genes; *Notch1* and *Bcl11b* that are mediated via the RAG complex and participate in lymphomagenesis (31,32). We examined genomic DNA from healthy thymocytes of WT RAG2 and RAG2^FS/FS^ to avoid any effects of p53 deficiency.

**Figure 4.**
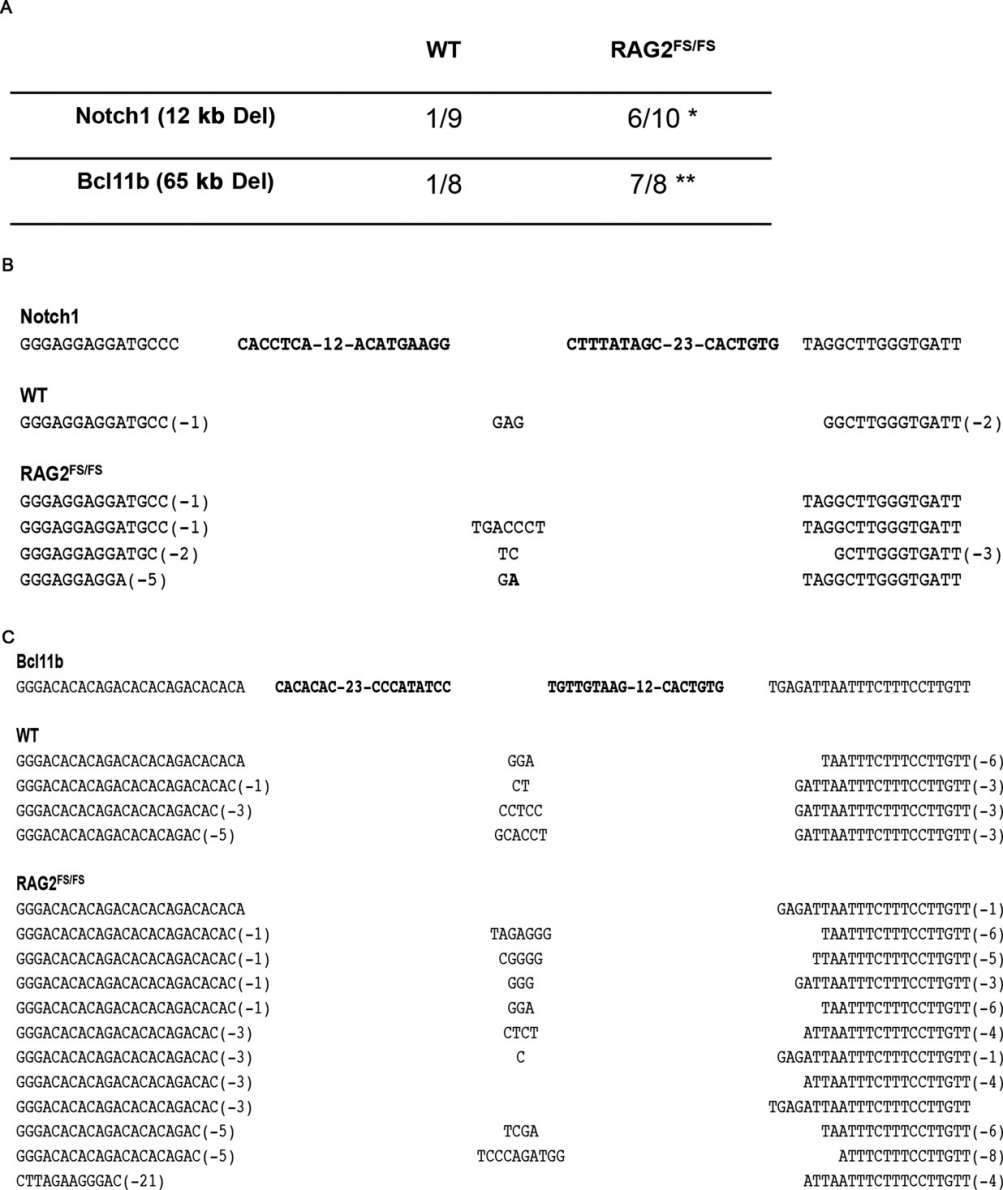
Intragenic deletions in Notch1 and Bcl11b. Genomic DNA from thymocytes of indicated genotypes was used in nested PCR to detect a 12 and 6 5kb deletions in Notch1 and Bcl11b genes, respectively. (**A**) Comparison between WT and RAG^FS/FS^ mice. *P < 0.03, **P < 0.003. Sequence analysis of the PCR products for Notch1 (**B**) and Bcl11b (**C**). First line represents the germline sequence with bold letters indicating the cRSS from each side. Capital letters in the middle of the junction are N nt. Bold capital letters are P nt. WT, *n* = 1; RAG2^FS/FS^, *n* = 2–4.

RAG2^FS/FS^ mice were significantly more efficient at recombining both the *Notch1* (*P* < 0.03) and *Bcl11b* (*P* < 0.003) genes than WT RAG2 (Figure 4A). Junction sequences were not qualitatively different between the two genotypes which is consistent with our finding that coding joints cannot be unambiguously assigned to either repair pathway (Figure 4B and C). The increased usage of cRSSs by the FS allele may provide an additional mechanism by which the lymphomas in RAG2^FS/FS^;p53^−/−^ mice evolve.

## DISCUSSION

We used two knock-in mouse models, RAG2^FS/FS^ and RAG2^del352/del352^, to demonstrate that in both c-NHEJ-proficient and c-NHEJ-deficient animals, removing RAG2's C-terminus allows aberrant use of a-NHEJ to join physiologic, chromosomal DSBs. These data provide the first evidence that pathway choice operates during normal V(D)J recombination *in vivo.* Is pathway choice physiologically relevant in c-NHEJ-proficient animals? Our data suggest that defects in pathway choice may explain, at least in part, the genomic instability seen in lymphomas from RAG2^FS/FS^;p53^−/−^ mice. However, until a-NHEJ is better defined we cannot rule out involvement of c-NHEJ in such rearrangements.

Additional evidence that RAG2's C-terminus is important for controlling repair pathway choice is provided by analysis of N nucleotide addition. Previous work showing that junctions formed in the absence of Ku80 were devoid of N additions indicated that Ku80 is important for recruiting terminal deoxynucleotidyl transferase (TdT) to DSBs (47–49). This does not appear to be the case in the presence of RAG2 C-terminal truncations where coding joints from double mutant mice displayed N regions. We speculate that this may be another feature of abrogated pathway choice control, in which TdT has unregulated access to RAG-mediated DSBs in the absence of RAG2's C-terminus. This is supported by our observation that in RAG2^FS/FS^ mice, N regions are significantly more prevalent at signal joints (35% in RAG2^FS/FS^ mice versus 20% in WT, *P* < 0.006).

Our analysis of aberrant junctions in mice bearing RAG2 C-terminal truncation also revealed a propensity for events involving cRSS, some of which involve known oncogenes. Similar events were recently reported in human B-ALL samples analyzed by next generation sequencing (50), indicating that these kinds of events can underlie human malignancies, even in the context of WT RAG2. Our data suggest that, while a-NHEJ may contribute to lymphomagenesis in this model, aberrant recognition of cRSS also may play an important role.

Because RAG2 C-terminal mutants allow joining by a-NHEJ, characteristics of chromosomal a-NHEJ can be studied in both c-NHEJ-proficient and c-NHEJ-deficient animals. Surprisingly, coding joints repaired by a-NHEJ do not exhibit features commonly associated with a-NHEJ, even in the absence of Ku80. The resemblance of a-NHEJ repair to that of c-NHEJ implies that the former might be more prevalent than appreciated and can, like c-NHEJ, repair physiological DSBs in a nonmutagenic fashion. This discovery suggests that we must exercise caution when interpreting features of DNA rearrangements in sequenced tumor genomes. Moreover, the unified sequence features of these junctions (e.g. the lack of a subset of junctions bearing excessive deletions, insertions or microhomologies) imply that they may be formed by a single repair pathway, rather than through a hodgepodge of joining activities. Our data have another important implication, which is that alternatives to c-NHEJ cannot be presumed to have an impact on junction structure. Only upon close scrutiny, we could detect any difference between the Ku80-independent coding joints and those generated in WT mice (ADRs, discussed above). Thus, either many repair factors are shared between c-NHEJ and alternative joining mechanisms, or the alternative pathway used to join coding ends is organized to call up a similar compilation of different enzymes that pretty nearly reconstruct the products of c-NHEJ. We are not the first to question the existence of a single, well-defined alternative NHEJ pathway (6,40), however, this study contributes a definitive illustration of the lack of consistency between different systems.

The striking finding that most coding joints have normal structures is in apparent conflict with both the signal joints recovered in this study as well as with results from our cell-based assay in which the RAG2FS mutant allows coding joints bearing excessive deletions and microhomologies to be formed on an extrachromosomal substrate (15). These apparent discrepancies may be the consequence of a context-dependent aspect of a-NHEJ. There are obvious differences in the requirements in each case: signal ends prior to joining have blunt-ended termini, coding ends need to undergo several processing steps before they can become ligated. There is evidence that the structure of the DNA ends at a DSB can affect their resolution (51). An extrachromosomal substrate can reflect much about physiological joining, however, there can be a difference between end-joining where cut ends are closely linked and may be able to collide at random, versus ends in the chromosome, where a break must be somehow bridged and stabilized for joining. Lastly, the sequence environment surrounding the DSBs may also influence joining. In Ig class switch recombination, the almost invariant appearance of microhomology at junctions produced by a-NHEJ may be attributed to the repetitive nature of the switch regions (8,52). This might also be the case in our extrachromosomal substrate, where a substantial (9 nt) microhomology is present near the break points (15). In antigen receptor loci, which lack this repetitive sequence environment, such repair might not be similarly favored.

In conclusion, our analysis of the role of the RAG C-terminus shows that it is critical for repair pathway choice *in vivo*, reveals new features of a-NHEJ, and is a critical departure point for further work. Previous mutational analysis of the C-terminus of RAG2 identified the evolutionarily conserved ‘acidic hinge’ as having a strong effect on coding joint outcomes scored in the extrachromosomal a-NHEJ assay (22). The same acidic hinge mutants also caused genomic instability in a pre-B cell line, implying physiologic relevance (22), in agreement with the results reported here. Whether the V(D)J joining system refines the outcome of joining by controlling where and when cleavage occurs (i.e. ‘context’) and by handing off the coding ends to defined pathways, and how this is achieved at a mechanistic level will be relevant to unraveling the mechanisms responsible for preserving genomic integrity during V(D)J recombination, and may illuminate end-joining metabolism in general.

## SUPPLEMENTARY DATA

Supplementary Data are available at NAR Online.

SUPPLEMENTARY DATA
