# Supplementary material for: RAG2 mutants alter DSB repair pathway choice in vivo and illuminate the nature of ‘alternative NHEJ’
Source: Nucleic Acids Res. 2014 Apr 20;42(10):6352–64. doi: 10.1093/nar/gku295 (PMC4041462; doi:10.1093/nar/gku295)
Supplement: SUPPLEMENTARY DATA [file supp_42_10_6352__index.html]

RAG2 mutants alter DSB repair pathway choice in vivo and illuminate the nature of ‘alternative NHEJ’ — RAG2 mutants alter DSB repair pathway choice in vivo and illuminate the nature of ‘alternative NHEJ’ — SUPPLEMENTARY DATA 

# RAG2 mutants alter DSB repair pathway choice *in vivo* and illuminate the nature of ‘alternative NHEJ’

## SUPPLEMENTARY DATA

**Files in this Data Supplement:**

- SUPPLEMENTARY DATA
