# Supplementary material for: RAG2 mutants alter DSB repair pathway choice in vivo and illuminate the nature of ‘alternative NHEJ’
Source: Nucleic Acids Res. 2014 Apr 20;42(10):6352–64. doi: 10.1093/nar/gku295 (PMC4041462; doi:10.1093/nar/gku295)
Supplement: SUPPLEMENTARY DATA [file supp_gku295_SI_index_Gigi_Roth_april_11_2014.pdf]

Supplementary Information

Figure S1. Generation of RAG2$^{FS/FS}$ knock-in mice.

A. RAG2FS has the highest a-NHEJ activity in-vitro. 293T cells were transiently transfected with full length Rag1, the different Rag2 mutants and the indicated substrate. Recombination was measured 48h post transfection by FACS analysis. Calculations are fold increase over full length Rag2 (RAG2). A. Recombination with RSSs in deletion configuration; RAG2, RAG2Core (a.a1-383) RAG2FS - Frame-shift mutation at amino acid 361, DDE- inactive Rag1. Absolute recombination for RAG2 28%±12. B. Recombination with a-NHEJ substrate (15). * p<0.0001 vs RAG2, **p<0.001 vs RAG2Core. RAG2 absolute recombination is 0.5%±0.25.

B. Mice were generated in inGenious Targeting Laboratory Inc. A~11.5kb region used to generate the targeting vector was first sub cloned from a positively identified C57/Bl6 BAC clone. Two types of mutations were generated in exon 3 utilizing overlap extension PCR. The first mutation comprised deletion of base 1082 (T) to generate a frameshift. The second mutation is located 67bp 3' of the T deletion and comprised replacement of the last 435bp of coding sequence with the sequence- AAGCGGCCGCGACTCTAG followed by the 3' UTR sequence. First, primers located 5' and 3' to two unique Kpn1 (K) sites that flank the location of the mutations were used to amplify a 1.6kb fragment. The mutations were introduced into this fragment, which was then reintroduced back into the construct via ligation into the Kpn1 sites, thus replacing the wild type Kpn1 fragment with the mutated Kpn1 fragment. The Neo cassette is inserted 228bps 5 to the ATG in exon 3 using Red/ET recombineering technology with a short homology arm that extends ~1.5kb 5' to the Neo cassette. The total size of the targeting construct (including the backbone vector) is 15.3kb. C. Identified F1 heterozygous mice were crossed with Ella-Cre transgenic mice to delete the floxed Neo cassette in vivo. An additional cross between the +/F mice and wild type mice eliminated the transgene. Subsequent mating of Cre-F2 (RAG2+/FS) generated littermates with the wild type (+/+), heterozygous (RAG2$^{+/FS}$) and homozygous (RAG2$^{FS/FS}$) genotypes for analysis. Genotyping analysis was done using the SQ1-GGAGACTCCTGACTGGACCCTCAG and DL1-GATTCAGAGAGCAATATACCT primers. D. Sequence analysis of tail and liver DNA showed an additional amino acid change at T296M that occurred during the targeting. Nevertheless, this change did not affect the FS mutant functionality in our cell system assay allowing us to still use it as a mouse model to investigate our questions.

Figure S2. T and B cell development in RAG2$^{FS/FS}$ knock-in mice.

A. Thymocytes from mice of the indicated genotype were stained with antiCD8-PE-CY7, antiCD4-APC-CY7, antiCD25-APC and antiCD44-PE. B. Summary table of the different B and T cell

populations from 6-8 week old mice n=7. RAG KO ($RAG^{-/-}$) are combined analysis of Rag1 and Rag2 KO n=4. B220+ cells in the BM are gated on IgM-. *p<0.05 vs WT.

Figure S3. Signal joints sequences from WT and $RAG2^{FS/FS}$ mice

A. SJ from Vβ14-Dβ1. B. SJ from Vδ5-Dδ2. C. SJ from Vβ8.3-Dβ1.1 D. SJ from Vβ10-Dβ1. Germ-line sequence of each locus is shown at the top. Capital letters indicate the RSS and small letters were indicted are coding end region. Capital letters in the middle of the junction indicate N nt, deletions are indicated in parentheses, small letter indicates sequences from the coding region (miscleavage), capital bold italics are microhomology and underlined blue are open shut junctions.

Figure S4. Coding joint sequences from WT and $RAG2^{FS/FS}$ mice. Whole thymocytes or BM cells from 2-3 mice were analyzed by PCR amplification of the indicated loci followed by Topo cloning. Germ line sequence is indicated at the top of each locus. Capital letters in the middle of the junction indicate N nt, capital bold are P nt, deletions are indicated in parentheses, small letter indicates sequences from the coding region (miscleavage). A. Vβ6/7/8-Jβ2 B. Vβ10-Jβ2.1 C. Vβ14-J1.1. Blue and red represents the ADRs D. Dβ2-Jβ2 (bold small letters represent the 12 RSS) E. Vh1783-Jh4. F.VH CDR3 length distribution analyzed by CDR3 spectratyping on spleen DNA (54). CDR3 length (bp) is plotted for WT and $RAG2^{FS/FS}$ mice. Each symbol represents a single rearrangement of J606.1-JH2 (left panel) or J558.82-JH2 (right panel). Each column is an individual mouse of a given genotype (n=3 per genotype). Horizontal bars indicate the median CDR3 length for all of the rearrangements sampled in a single mouse.

Figure S5. $RAG2^{FS/FS}$ interchromosomal rearrangements within the antigen receptor loci.

A. Scheme of the germ-line configuration of TCR beta and delta and the predicted products upon interchromosomal rearrangements. Rectangles represent coding gene segments; triangles represent RSS (Filled -12RSS, open - 23 RSS). The arrows indicate nested PCR primers. B. Sequence analysis of purified PCR products (n=4 $RAG2^{FS/FS}$ mice). Capital letters at the middle of the junction represents N nt, Bold italic are microhomology, deletions are indicated in parentheses and small letters are Sanger sequence that did not align to mouse mm9 database.

Figure S6. Generation of Adjacent Direct Repeats -ADRs

A. Scheme showing how small ADRs (adjacent direct repeats) might be generated. The first step may or may not be a mechanistic constraint however it is necessary in the identification of ADRs. We show homologies between ends having been generated by TdT or perhaps another

polymerase (53). Terminal homologies can also be revealed between two ends by resection but were excluded in our analysis because prior existing sequences between ends that can generate ADRs are indistinguishable from simple direct joining products and difficult to score. The next step is stabilizing end-to-end interaction via complementary bases in the two single strand extensions compensating for the lack of Ku80. We suggest that annealing occurs near or at the termini. For the sake of parsimony, we have depicted Fen-1 as the flap removal factor prior to the gap-filling and strand displacement steps. ADRs are then generated when a gap-filling polymerase with strand displacement activity is present. This could be supplied by pol lamda (39), which has sufficient strand displacement activity in vitro with the cooperation of Fen-1, to invade three or four bp into a duplex, or by pol beta, which display stronger strand displacement on its own (38). The latter is not a known participant in cNHEJ, but is also stimulated by Fen-1 *in vitro* (39). Following polymerization/strand displacement of one end the second detached end could be either filled in by 'conventional' extension and then ligated or alternatively, ligated and then filled in.

B. Summary of the number of junctions with ADRs detected in our study and by re-examination of published data (23,25). The ADRs are indicated in Blue-Red in Fig.S4. nd stands for Not Done.

Figure S7. RAG2del352 mice junctions' analysis.

A. Diagram of the RAG2del352 allele. A change in RAG2 sequence after amino acid 352 originating from the targeting vector is indicated in red. B. Signal joint analysis from different TCR loci n=2-6. (Analysis was preformed as specified in Table 1). C. Dδ2-Dβ1 interchromosomal rearrangements from healthy thymocytes n=3 (Details as specified in Fig.S5). D. $RAG2^{del352/del352}$;$Ku80^{-/-}$ antigen receptor rearrangements from the indicated loci n=1-3. (Annotations are as given in Fig.2).

Figure S8. $RAG2^{FS/FS}$;$P53^{-/-}$ lymphomas analysis.

A. FACS analysis of representative wild-type thymus and $RAG2^{FS/FS}$;$p53^{-/-}$ thymic lymphomas. B. Endogenous antigen receptor rearrangements detected by whole genome sequencing. Seven endogenous genomic rearrangements detected in tumor 13422. All rearrangements were in the coding end configuration. End1 represents the coding end of a 23RSS; End 2 represents the coding end of 12RSS. Capital letters in the middle are N nt and Bold are P nt.

Supplementary Methods

V(D)J recombination assay

The 293T cell line was grown in DMEM supplemented with fetal bovine serum (10%), non-essential amino acids and penicillin-streptomycin. Cells were grown at 37 °C in the presence of 5% CO2. To assess V(D)J recombination, 0.5 µg of the indicated murine Rag1, murine Rag2 and recombination substrate were transfected into cells using a FuGENE 6:DNA ratio of 3:1. Forty-eight hours after transfection cells were harvested and fluorescent intensity was measured using BD LSR II for FACS readout.

Spectratyping

Immunoglobulin heavy chain repertoire analysis was performed using CDR3 spectratyping, as described (54). Briefly, genomic DNA from splenocytes was purified using PureGene and amplified using the J606.1 and the J558.85 VH primers and a fluorescent labeled JH2 reverse primer. 2 µL of PCR product per reaction were resolved by capillary electrophoresis on an ABI 3100 analyzer (Applied Biosciences Inc). Peak scanner v. 1.0 was used to generate and analyze the spectratypes (Applied Biosciences Inc) as described (54).

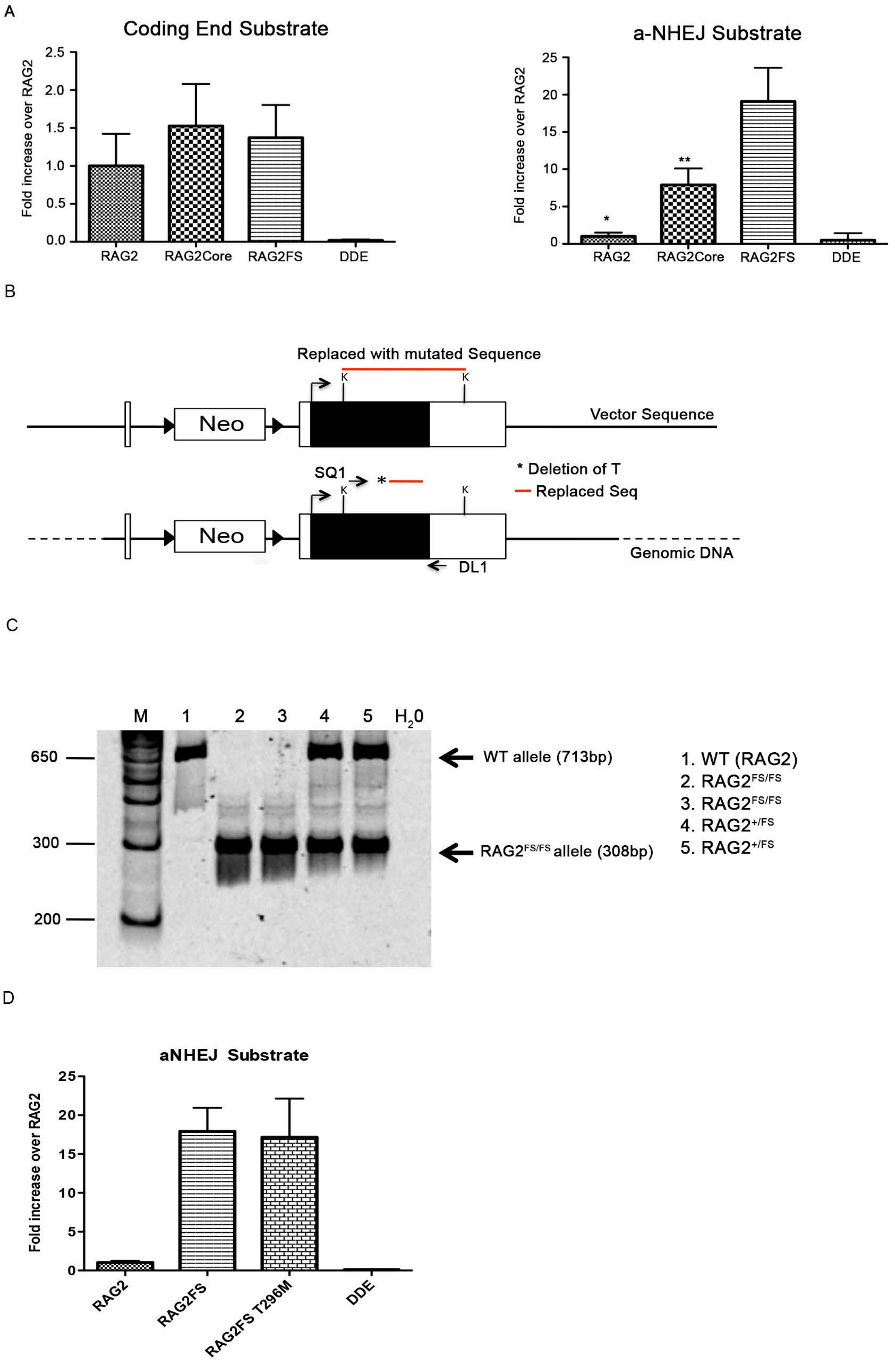
A
Coding End Substrate
Fold increase over RAG2
2.5
2.0
1.5
1.0
0.5
0.0
RAG2
RAG2Core
RAG2FS
DDE
a-NHEJ Substrate
Fold increase over RAG2
25
20
15
10
5
0
*
**
RAG2
RAG2Core
RAG2FS
DDE
B
Replaced with mutated Sequence
K
K
Neo
Vector Sequence
SQ1
*
* Deletion of T
— Replaced Seq
K
K
Neo
Genomic DNA
DL1
C
M
1
2
3
4
5
H2O
650
300
200
WT allele (713bp)
RAG2FS/FS allele (308bp)
1. WT (RAG2)
2. RAG2FS/FS
3. RAG2FS/FS
4. RAG2+/FS
5. RAG2+/FS
D
aNHEJ Substrate
Fold increase over RAG2
25
20
15
10
5
0
RAG2
RAG2FS
RAG2FS T296M
DDE

Fig. S1

A. Thymus

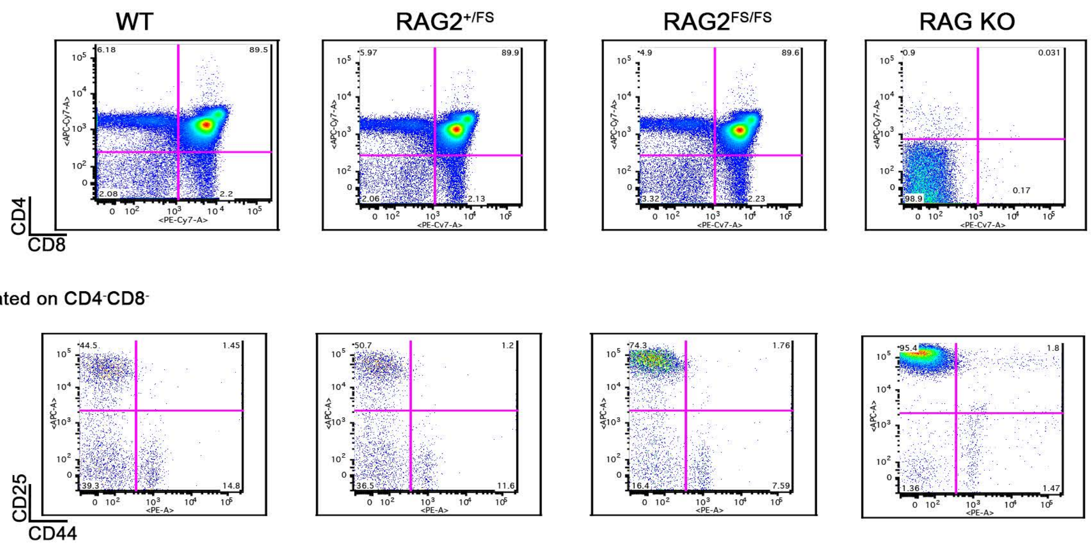

B.

Thymus	% CD4⁺CD8⁺	%CD4⁻CD8⁻	% CD44⁻CD25⁺	% CD44⁻CD25⁻
WT	88.37±1.59	1.85±0.46	38.92±11.81	46.10±11.73
RAG2$^{+/FS}$	87.74±2.96	2.44±0.45	42.06±12.31	42.63±14.48
**RAG2$^{FS/FS}$**	**88.20±2.25**	**3.02±0.63***	**65.09±6.23***	**22.42±6.67***
RAG$^{-/-}$	0.06±0.00	99.10±0.28	95.5±1.13	1.71±1.06

BM	% IgM⁺	% IgM⁻	% B220⁺CD43⁺	% B220⁺CD43⁻
WT	42.2±13.7	52.1±17.1	16.3±3.5	51.6±15.5
RAG2$^{+/FS}$	43.0±5.1	52.3±9.9	14.4±4.5	49.6±15.6
**RAG2$^{FS/FS}$**	**31.6±7.4**	**63.9±13.2**	**24.0±2.7***	**39.1±11.4**
RAG$^{-/-}$	1±0	99±0	16.8±1.9	1.7±0.25

Spleen	% CD4⁺	%CD8⁺	% B220⁺IgM⁺
WT	17.2±1.5	7.7±1.4	35±3.1
**RAG2$^{FS/FS}$**	**14.7±1.9**	**8.2±1.2**	**29.3±4.4**
RAG$^{-/-}$	2.5±1.7	0.4±0.4	0.06±0.09

Fig. S2

A

Vβ14		Dβ1
GCACAGATGTCTGCCCCACCCTACTCAGTGTGagact		ccctgtcccCACAATGTTACAGCTTTATACAAAAAAGG

WT

GCACAGATGTCTGCCCCACCCTACTCAGTGTG	ACCCCCT	CACAATGTTACAGCTTTATACAAAAAAGG
GCACAGATGTCTGCCCCACCCTACTCAGTGTG	GACGG	CACAATGTTACAGCTTTATACAAAAAAGG
GCACAGATGTCTGCCCCACCCTACTCAGTGTG	CCAG	CACAATGTTACAGCTTTATACAAAAAAGG
GCACAGATGTCTGCCCCACCCTACTCAGTGTG	CCC	CACAATGTTACAGCTTTATACAAAAAAGG
GCACAGATGTCTGCCCCACCCTACTCAGTGTG	GTC	CACAATGTTACAGCTTTATACAAAAAAGG
GCACAGATGTCTGCCCCACCCTACTCAGTGTG	CT	CACAATGTTACAGCTTTATACAAAAAAGG
GCACAGATGTCTGCCCCACCCTACTCAGTGTG	GG	CACAATGTTACAGCTTTATACAAAAAAGG
GCACAGATGTCTGCCCCACCCTACTCAGTGTG	CC	CACAATGTTACAGCTTTATACAAAAAAGG
GCACAGATGTCTGCCCCACCCTACTCAGTGTG	T	CACAATGTTACAGCTTTATACAAAAAAGG
GCACAGATGTCTGCCCCACCCTACTCAGTGTG	G	CACAATGTTACAGCTTTATACAAAAAAGG
GCACAGATGTCTGCCCCACCCTACTCAGTGTGagact		CACAATGTTACAGCTTTATACAAAAAAGG

RAG2$^{FS/FS}$

GCACAGATGTCTGCCCCACCCTACTCAGTGTG	GCCT	CACAATGTTACAGCTTTATACAAAAAAGG
GCACAGATGTCTGCCCCACCCTACTCAGTGTG	ACCG	CACAATGTTACAGCTTTATACAAAAAAGG
GCACAGATGTCTGCCCCACCCTACTCAGTGTG	CCC	CACAATGTTACAGCTTTATACAAAAAAGG
GCACAGATGTCTGCCCCACCCTACTCAGTGTG	GG	CACAATGTTACAGCTTTATACAAAAAAGG
GCACAGATGTCTGCCCCACCCTACTCAGTGTG	TC	CACAATGTTACAGCTTTATACAAAAAAGG
GCACAGATGTCTGCCCCACCCTACTCAGTGTG	CT	CACAATGTTACAGCTTTATACAAAAAAGG
GCACAGATGTCTGCCCCACCCTACTCAGTGTG	GA	CACAATGTTACAGCTTTATACAAAAAAGG
GCACAGATGTCTGCCCCACCCTACTCAGTGTG	GT	CACAATGTTACAGCTTTATACAAAAAAGG
GCACAGATGTCTGCCCCACCCTACTCAGTGTG	G	CACAATGTTACAGCTTTATACAAAAAAGG
GCACAGATGTCTGCCCCACCCTACTCAGTGTG	T	CACAATGTTACAGCTTTATACAAAAAAGG
GCACAGATGTCTGCCCCACCCTACTCAGTGTG	350bp	CACAATGTTACAGCTTTATACAAAAAAGG
GCACAGATGTCTGCCCCACCCTACTCAGTGTG	GGGT	TTACAGCTTTATACAAAAAAGG(-7)
GCACAGATGTCTGCCCCACCCTACTCAGTGTG	G	AATGTTACAGCTTTATACAAAAAAGG(-3)
GCACAGATGTCTGCCCCACCCTACTCAGTGTG		ATGTTACAGCTTTATACAAAAAAGG(-4)
GCACAGATGTCTGCCCCACCCTACTCAGTG(-2)	CGCC	CACAATGTTACAGCTTTATACAAAAAAGG
GCACAGATGTCTGCCCCACCCT(-10)		ATGTTACAGCTTTATACAAAAAAGG(-4)
GCACAGATGTCTGCCCCACC(-12)		TTACAGCTTTATACAAAAAAGG(-7)
GCACAGATGTCT***GC***(-18)		TTTATACAAAAAAGG(-14)
TGT***GGT***(-36)		CTACCC(-43)
TCCCTAG(-81)		CTGC(-79)
GCACAGATGTCTGCCCCACCCTACTCAGTGTGagact	CTG	ccctgtcccCACAATGTTACAGCTTTATACAAAAAAGG
GCACAGATGTCTGCCCCACCCTACTCAGTGTGagactc	GGT	cccctgtcccCACAATGTTACAGCTTTATACAAAAAAGG
GCACAGATGTCTGCCCCACCCTACTCAGTGTGagact		cccctgtcccCACAATGTTACAGCTTTATACAAAAAAGG

B

Vδ5		Dδ2
GGTTTGGGTACAGGCTCCCTGGGCACCTGCACCACAGTG		ctcgtatccctccgatCACGGTGCTACAGAGCTTTGCAAAAACC

WT

GGTTTGGGTACAGGCTCCCTGGGCACCTGCACCACAGTG	CTT	CACGGTGCTACAGAGCTTTGCAAAAACC
GGTTTGGGTACAGGCTCCCTGGGCACCTGCACCACAGTG	GTT	CACGGTGCTACAGAGCTTTGCAAAAACC
GGTTTGGGTACAGGCTCCCTGGGCACCTGCACCACAGTG	GGT	CACGGTGCTACAGAGCTTTGCAAAAACC
GGTTTGGGTACAGGCTCCCTGGGCACCTGCACCACAGTG	GCCATCA	CACGGTGCTACAGAGCTTTGCAAAAACC
GGTTTGGGTACAGGCTCCCTGGGCACCTGCACCACAGTG	GA	CACGGTGCTACAGAGCTTTGCAAAAACC
GGTTTGGGTACAGGCTCCCTGGGCACCTGCACCACAGTG	GGAT	CACGGTGCTACAGAGCTTTGCAAAAACC
GGTTTGGGTACAGGCTCCCTGGGCACCTGCACCACAGTG	GG	CGGTGCTACAGAGCTTTGCAAAAACC(-2)
GGTTTGGGTACAGGCTCCCTGGGCACCTGCACCACAG(-2)	GGCG	CACGGTGCTACAGAGCTTTGCAAAAACC
GGTTTGGGTACAGGCTCCCTGGGCACCTGCACCACAG(-2)	CTT	ACGGTGCTACAGAGCTTTGCAAAAACC(-1)

RAG2$^{FS/FS}$

GGTTTGGGTACAGGCTCCCTGGGCACCTGCACCACAGTG	CCCCC	CACGGTGCTACAGAGCTTTGCAAAAACC
GGTTTGGGTACAGGCTCCCTGGGCACCTGCACCACAGTG	GGGG	CACGGTGCTACAGAGCTTTGCAAAAACC
GGTTTGGGTACAGGCTCCCTGGGCACCTGCACCACAGTG	TT	CACGGTGCTACAGAGCTTTGCAAAAACC
GGTTTGGGTACAGGCTCCCTGGGCACCTGCACCACAGTG	GT	GGTGCTACAGAGCTTTGCAAAAACC(-3)
GGTTTGGGTACAGGCTCCCTGGGCACCTGCACCACAGTG	TCCCAGT	TGCTACAGAGCTTTGCAAAAACC(-5)
GGTTTGGGTACAGGCTCCCTGGGCACCTGCACCACAG(-2)		CGGTGCTACAGAGCTTTGCAAAAACC(-2)
GGTTTGGGTACAGGCTCCCTGGGCACCTGCACCACAG(-2)	GG	GCTACAGAGCTTTGCAAAAACC(-6)
GGTTTGGGTACAGGCTCCCTGGGCACCTGCACCACAG(-2)	AGGGGGCCT	CTACAGAGCTTTGCAAAAACC(-7)
GGTTTGGGTACAGGCTCCCTGGGCACCTGCACCACA(-3)	A	GGTGCTACAGAGCTTTGCAAAAACC(-3)
GGTTTGGGTACAGGCTCCCTGGGCACCTGCACC(-6)	CCTCGT	TGCTACAGAGCTTTGCAAAAACC(-5)
GGTTTGGGTACAGGCTCCCTGGGCACCTGC(-10)		CCTGGGCTTT(-31)
GGTTTGGGTACAGGCTCCCTGGGCAC(-13)	AA	ACGGTGCTACAGAGCTTTGCAAAAACC(-1)
GGTTTGGGTACAGGC***TCCCTGGGC***(-15)		TTT(-38)
GGTTTGGGTACAGGC(-24)		CTACAGAGCTTTGCAAAAACC(-7)
CAGGGG***TTTG***(-32)		TA(-42)
GG(-59)		CCCCAG(-62)
GGTTTGGGTACAGGCTCCCTGGGCACCTGCACCACA(-3)	CCAAG	ctcgtatccctccgatCACGGTGCTACAGAGCTTTGCAAAAACC
GGTTTGGGTACAGGCTCCCTGGGCACCTGCACCACA(-3)	ATTATGCGCG	gtatccctccg**TTATTT**TGCTACAGAGCTTTGCAAAAACC(-5)
GGTTTGGGTACAGGCTCCCTGGGCACCTGCACCAC(-4)	TAG	ctcgtatccctccg**TCTG**GTGCTACAGAGCTTTGCAAAAACC(-4)
GGTTTGGGTACAGGCTCCCTGGGCACCTGCACC(-6)	TAG	ctcgtatccctccgatCACGGTGCTACAGAGCTTTGCAAAAACC

Fig. S3

C

Vβ8.3		Dβ1.1
ACTTTCTGTGCAAAGGGGAGGAAGCCACACATCACTGTG	GCCCCCTGTCCC	cccctgtcccCACAATGTTACAGCTTTATACAAAAAAG

WT

ACTTTCTGTGCAAAGGGGAGGAAGCCACACATCACTGTG	AA	CACAATGTTACAGCTTTATACAAAAAAG
ACTTTCTGTGCAAAGGGGAGGAAGCCACACATCACTGTG	GG	CACAATGTTACAGCTTTATACAAAAAAG
ACTTTCTGTGCAAAGGGGAGGAAGCCACACATCACTGTG	GG	CACAATGTTACAGCTTTATACAAAAAAG
ACTTTCTGTGCAAAGGGGAGGAAGCCACACATCACTGTG	G	CACAATGTTACAGCTTTATACAAAAAAG
ACTTTCTGTGCAAAGGGGAGGAAGCCACACATCACTGTG	C	CACAATGTTACAGCTTTATACAAAAAAG
ACTTTCTGTGCAAAGGGGAGGAAGCCACACATCACTGTG	CCCCCCA	AATGTTACAGCTTTATACAAAAAAG(-3)
ACTTTCTGTGCAAAGGGGAGGAAGCCACACATCACTGTG	TCATAG	TGTTACAGCTTTATACAAAAAAG(-5)
ACTTTCTGTGCAAAGGGGAGGAAGCCACACATCACTG(-2)	CGGG	CACAATGTTACAGCTTTATACAAAAAAG

RAG$^{2FS/FS}$

ACTTTCTGTGCAAAGGGGAGGAAGCCACACATCACTGTG	CTCCCTC	CACAATGTTACAGCTTTATACAAAAAAG
ACTTTCTGTGCAAAGGGGAGGAAGCCACACATCACTGTG	TCCTCC	CACAATGTTACAGCTTTATACAAAAAAG
ACTTTCTGTGCAAAGGGGAGGAAGCCACACATCACTGTG	GGGGA	CACAATGTTACAGCTTTATACAAAAAAG
ACTTTCTGTGCAAAGGGGAGGAAGCCACACATCACTGTG	GGC	CACAATGTTACAGCTTTATACAAAAAAG
ACTTTCTGTGCAAAGGGGAGGAAGCCACACATCACTGTG	GGT	CACAATGTTACAGCTTTATACAAAAAAG
ACTTTCTGTGCAAAGGGGAGGAAGCCACACATCACTGTG	CC	CACAATGTTACAGCTTTATACAAAAAAG
ACTTTCTGTGCAAAGGGGAGGAAGCCACACATCACTGTG	CC	CACAATGTTACAGCTTTATACAAAAAAG
ACTTTCTGTGCAAAGGGGAGGAAGCCACACATCACTGTG	GG	CACAATGTTACAGCTTTATACAAAAAAG
ACTTTCTGTGCAAAGGGGAGGAAGCCACACATCACTGTG	C	cccCACAATGTTACAGCTTTATACAAAAAAG
ACTTTCTGTGCAAAGGGGAGGAAGCCACA(-10)	TT	ccctgtcccCACAATGTTACAGCTTTATACAAAAAAG
ACTTTCTGTGCAAAGGGGAGGAAGCCAC(-11)	TCCTTA	cccctgtcccCACAATGTTACAGCTTTATACAAAAAAG
ACTTTCTGTGCAAAGGGGAGGAAGCC(-13)	GCCT	cccctgtcccCACAATGTTACAGCTTTATACAAAAAAG
ACTTC(-72)	A	ctgtcccCACAATGTTACAGCTTTATACAAAAAAG

D

Vβ10		Dβ1.1
TGGGTTTGTGCACAGGGAAACAGTGACTCTGCACAACTGTGtcttagctgc		gcccctgtcccCACAATGTTACAGCTTTATACAAAAAAGGACCC

WT

TGGGTTTGTGCACAGGGAAACAGTGACTCTGCACAACTGTG	AGCGT	CACAATGTTACAGCTTTATACAAAAAAGGACCC
TGGGTTTGTGCACAGGGAAACAGTGACTCTGCACAACTGTG	GGT	CACAATGTTACAGCTTTATACAAAAAAGGACCC
TGGGTTTGTGCACAGGGAAACAGTGACTCTGCACAACTGTG	CC	CACAATGTTACAGCTTTATACAAAAAAGGACCC
TGGGTTTGTGCACAGGGAAACAGTGACTCTGCACAACTGTG	GC	CACAATGTTACAGCTTTATACAAAAAAGGACCC
TGGGTTTGTGCACAGGGAAACAGTGACTCTGCACAACTGTG	GC	CACAATGTTACAGCTTTATACAAAAAAGGACCC
TGGGTTTGTGCACAGGGAAACAGTGACTCTGCACAACTGTG	GG	CACAATGTTACAGCTTTATACAAAAAAGGACCC

RAG$^{2FS/FS}$

TGGGTTTGTGCACAGGGAAACAGTGACTCTGCACAACTGTG	26bp	ccctgtcccCACAATGTTACAGCTTTATACAAAAAAGGACCC
TGGGTTTGTGCACAGGGAAACAGTGACTCTGCACAACTGTG	GGTCACG	ATGTTACAGCTTTATACAAAAAAGGACCC(-4)
TGGGTTTGTGCACAGGGAAACAGTGACTCTGCgCAACTGTG	GGACT	CACAATGTTACAGCTTTATACAAAAAAGGACCC
TGGGTTTGTGCACAGGGAAACAGTGACTCTGCACAACTGTG	AC	CACAATGTTACAGCTTTATACAAAAAAGGACCC
TGGGTTTGTGCACAGGGAAACAGTGACTCTGCACAACTGTGtcttagctgc	G	gcccctgtcccCACAATGTTACAGCTTTATACAAAAAAGGACCC
TGGGTTTGTGCACAGGGAAACAGTGACTCTGCACAACTGTG	C	CACAATGTTACAGCTTTATACAAAAAAGGACCC
TGGGTTTGTGCACAGGGAAACAGTGACTCTG***CACAA***(-5)		TGTTACAGCTTTATACAAAAAAGGACCC(-5)

Fig.S3

A

		Dβ1/2		
Vβ8.2	GTACTTCTGTGCCAGCGGTGATG	GGGACAGGGGGC	AGTCAAAACACCTTGTACTTTGGGCCA	Jβ2.4
Vβ8.1	TATATTTCTGTGCCAGCAGTGATG	GGGACTGGGGGGGC	AACCAAGACACCCAGTACTTGGGCCA	Jβ2.5
Vβ7	TGTACTTCTGTGCTAGCAGTTTATC		CTCCTATGAACAGTACTTCGGTCCCGG	Jβ2.7
Vβ6	TTTTTCTCTGTGCCAGCAGTATAG			

WT

GTACTTCTGTGCCAGCGGTGATG	TC			AAGACACCCAGTACTTTGGGCCA(-4)
TTTTTCTCTGTGCCAGCAGTATAG		(-4)CTGGGG(-4)		GACACCCAGTACTTTGGGCCA(-6)
TATATTTCTGTGCCAGCAGTGATG	G	GGGACAGGG(-3)		AAGACACCCAGTACTTTGGGCCA(-4)
TATATTTCTGTGCCAGCAGTGATG	**C**	GGGGGG	AC	GAACAGTACTTCGGTCCCGG(-7)
TGTACTTCTGTGCTAGCAGTTTATC		(-4)CAGGG(-3)		ATGAACAGTACTTCGGTCCCGG(-5)
GTACTTCTGTGCCAGCGGTGAT(-1)			AATC	TATGAACAGTACTTCGGTCCCGG(-4)
GTACTTCTGTGCCAGCGGTGAT(-1)	A	(-6)GGGGGGGC	**GCC**GG	CACCCAGTACTTTGGGCCA(-8)
GTACTTCTGTGCCAGCGGTGAT(-1)		(-4)CTGGG(-5)	AA	GAACAGTACTTCGGTCCCGG(-7)
TTTTTCTCTGTGCCAGCAGTATA(-1)		(-5)AGGGG(-2)	A	ATGAACAGTACTTCGGTCCCGG(-4)
TTTTTCTCTGTGCCAGCAGTATA(-1)	CC	GGGGC	**G**	CCAAGACACCCAGTACTTTGGGCCA(-2)
TATATTTCTGTGCCAGCAGTGAT(-1)	C	(-4)CAGGGGG(-1)	GGTTAGC	ACAGTACTTCGGTCCCGG(-9)
GTACTTCTGTGCCAGCGGTGA(-2)		(-4)CTGGGGGGC	**GCC**GG	CCAAGACACCCAGTACTTTGGGCCA(-2)
TATATTTCTGTGCCAGCAGTGA(-2)		(-4)CTGGGGGGG(-1)		ATGAACAGTACTTCGGTCCCGG(-5)
TGTACTTCTGTGCTAGCAGTTTA(-2)		GGGGGC	G	ACCAAGACACCCAGTACTTTGGGCCA(-1)
GTACTTCTGTGCCAGCGGTG(-3)	T	(-4)CTGG(-6)	T**CT**	AGTCAAAACACCTTGTACTTTGGTGCG
GTACTTCTGTGCCAGCGGTG(-3)		GC	CCAA	TCAAAACACCTTGTACTTTGGTGCG(-2)
TGTACTTCTGTGCTAGCAGTTT(-3)		(-5)AGGGGGC	**G**	ACCAAGACACCCAGTACTTTGGGCCA(-1)
TGTACTTCTGTGCTAGCAGTT(-4)		(-3)ACAGGGG(-2)	T	CAAGACACCCAGTACTTTGGGCCA(-3)
TGTACTTCTGTGCTAGCAG(-6)	CC	(-2)GACAGGG(-3)	ATGG	GAACAGTACTTCGGTCCCGG(-7)
TGTACTTCTGTGCTAGCAG(-6)	CCT**CC**	GGGACTGGGGG(-3)	AT	CTATGAACAGTACTTCGGTCCCGG(-3)
TGTACTTCTGTGCTAGC(-8)		T		CAAGACACCCAGTACTTTGGGCCA(-3)
TGTACTTCTGTGCTAGC(-8)	T	(-5)TGGGG(-4)	A	AAGACACCCAGTACTTTGGGCCA(-4)
TGTACTTCTGTGCTA(-10)	CCCAG	(-5)TGGGGGG(-2)		ATGAACAGTACTTCGGTCCCGG(-5)

$RAG2^{FS/FS}$

GTACTTCTGTGCCAGCGGTGATG	**C**GT	(-4)CAGGG(-3)	TCCCC	AAGACACCCAGTACTTTGGGCCA(-4)
GTACTTCTGTGCCAGCGGTGATG	**C**	GG		GAACAGTACTTCGGTCCCGG(-7)
TATATTTCTGTGCCAGCAGTGATG	CAG	(-5)AGGG(-3)	ACATCT	AGACACCCAGTACTTTGGGCCA(-5)
TTTTTCTCTGTGCCAGCAGTATAG		(-4)ACGGG(-3)	TGGG	TATGAACAGTACTTCGGTCCCGG(-4)
TTTTTCTCTGTGCCAGCAGTATAG		(-3)ACTGGGGGG(-1)	ACGAGGA	GAACAGTACTTCGGTCCCGG(-7)
TTTTTCTCTGTGCCAGCAGTATAG		(-5)AGGGGG(-1)	G	TGAACAGTACTTCGGTCCCGG(-6)
GTACTTCTGTGCCAGCGGTGAT(-1)	T	(-4)CAGGGGG(-2)	GATAAG	CACCCAGTACTTTGGGCCA(-8)
GTACTTCTGTGCCAGCGGTGAT(-1)	CG	(-4)CTGGGGGG(-2)		ACCAAGACACCCAGTACTTTGGGCCA(-1)
GTACTTCTGTGCCAGCGGTGA(-2)	AGA	(-5)TGGGG(-4)	AT	TATGAACAGTACTTCGGTCCCGG(-4)
TATATTTCTGTGCCAGCAGTGA(-2)	C	GGGG	GATTGATCGGGG	ACCAAGACACCCAGTACTTTGGGCCA(-1)
TATATTTCTGTGCCAGCAGTGA(-2)	A	(-1)GGACTGGGGGGGC	TA	GAACAGTACTTCGGTCCCGG(-7)
TTTTTCTCTGTGCCAGCAGTAT(-2)	AAC	GGC	AG	AAGACACCCAGTACTTTGGGCCA(-4)
TGTACTTCTGTGCTAGCAGTTT(-3)	C	(-2)GACAG(-5)		CTCCTATGAACAGTACTTCGGTCCCGG
TGTACTTCTGTGCTAGCAGTTT(-3)	GTCT	(-4)CAGGG(-3)	ATGAG	GTACTTCGGTCCCGG(-12)
TGTACTTCTGTGCTAGCAGTTT(-3)		(-2)GACAGGGGGC	AGG	GACACCCAGTACTTTGGGCCA(-6)
TGTACTTCTGTGCTAGCAGTTT(-3)	G	AGGGGGC	**G**G	GAACAGTACTTCGGTCCCGG(-7)
TATATTTCTGTGCCAGCAGT(-4)	CCCT	(-2)GACAGGG(-3)	AGG	GAACAGTACTTCGGTCCCGG(-7)
TTTTTCTCTGTGCCAGCAGT(-4)	ACG	(-3)ACAGG(-4)		CTATGAACAGTACTTCGGTCCCGG(-3)
TTTTTCTCTGTGCCAGCAGT(-4)	GC	(-1)GGACTGGGGGGGC	**GT**	TGAACAGTACTTCGGTCCCGG(-6)
TGTACTTCTGTGCTAGCAGTT(-4)	C	GGGG		CAAAACACCTTGTACTTTGGTGCG(-3)
TGTACTTCTGTGCTAGCAGTT(-4)	C	(-3)ACTGGGG(-4)		AACCAAGACACCCAGTACTTGGGCCA
TTTTTCTCTGTGCCAGCAG(-5)		(-3)ACTGGGGGGGC	**GC**GGG	GACACCCAGTACTTTGGGCCA(-6)
TGTACTTCTGTGCTAGCAGT(-5)	CCT	(-3)ACAGGG(-3)		AACCAAGACACCCAGTACTTGGGCCA
TGTACTTCTGTGCTAGCAGT(-5)	AT**CC**	GGGACAGGGGGC	T	CACCCAGTACTTTGGGCCA(-8)
TGTACTTCTGTGCTAGCAGT(-5)		(-4)CTGG(-6)	ACAGT	TATGAACAGTACTTCGGTCCCGG(-4)
TGTACTTCTGTGCTAGCAGT(-5)	AC	(-2)GACTGGGGGGGC	**GC**TG	GAACAGTACTTCGGTCCCGG(-7)
TGTACTTCTGTGCTAGCAG(-6)	CTT	GGGACAGGG(-3)	A	GAACAGTACTTCGGTCCCGG(-7)
TGTACTTCTGTGCTAGCAG(-6)	CCCA	(-3)ACAGGG(-3)	TT	TGAACAGTACTTCGGTCCCGG(-6)
TGTACTTCTGTGCTAGCAG(-6)	CA	(-3)ACTGGGGGGGC	**GC**GGT	CCAAGACACCCAGTACTTTGGGCCA(-2)
TGTACTTCTGTGCTAGCAG(-6)	CCCC	GGGGG		CTATGAACAGTACTTCGGTCCCGG(-3)
TTTTTCTCTGTGCCAGCAGTATAGcacagtgga			A	CTATGAACAGTACTTCGGTCCCGG(-3)

Fig. S4

B

Vβ10		Dβ1/2		Jβ2.1
TCTCTGTGCCAGCAGCTAAGA		GGGACAGGGGGC<br>GGGACTGGGGGGGC		TAACTATGCTGAGCAGTTCTTCG

WT

TCTCTGTGCCAGCAGCTAAG(-1)	**C**	GGGAC		AACTATGCTGAGCAGTTCTTCG(-1)
TCTCTGTGCCAGCAGCTAA(-2)	AGG	(-4)CAGGGGG(-1)	**TTA**	TAACTATGCTGAGCAGTTCTTCG
TCTCTGTGCCAGCAGCTA(-3)	TT	(-5)TGGGGG(-3)		ATGCTGAGCAGTTCTTCG(-5)
TCTCTGTGCCAGCAGCTA(-3)	**CC**	GGGACAGGGGGC	**GC**	GCTGAGCAGTTCTTCG(-7)
TCTCTGTGCCAGCAGCTA(-3)	TCCT	(-2)GACA(-6)	ACGA	TATGCTGAGCAGTTCTTCG(-4)
TCTCTGTGCCAGCAGCTA(-3)		TGGGGGGG	GGT	ACTATGCTGAGCAGTTCTTCG(-2)
TCTCTGTGCCAGCAGCT(-4)		(-4)CTGGGGGGG(-1)	A	TATGCTGAGCAGTTCTTCG(-4)
TCTCTGTGCCAGCAGCT(-4)	CGCC	GGGC	CT	CTATGCTGAGCAGTTCTTCG(-3)
TCTCTGTGCCAGCAGCT(-4)	CCCGG	(-4)CTGGGGGGG(-1)		AACTATGCTGAGCAGTTCTTCG(-1)
TCTCTGTGCCAGCAGCT(-4)	T	(-2)GACTGGGGGG(-2)		AACTATGCTGAGCAGTTCTTCG(-1)
TCTCTGTGCCAGCAGC(-5)	GC	(-3)ACTGGGGGGGC	**GC**TGA	ATGCTGAGCAGTTCTTCG(-5)
TCTCTGTGCCAGCAG(-6)		(-3)ACTG(-7)	AG	AACTATGCTGAGCAGTTCTTCG(-1)
TCTCTGTGCCAGCAG(-6)	TCAAG	GGGACAGGGG(-2)	AGAG	ATGCTGAGCAGTTCTTCG(-5)
TCTCTGTGCCAGCAG(-6)	TC	(-1)GGACTGGGGGGG(-1)	G	CTATGCTGAGCAGTTCTTCG(-3)
TCTCTGTGCCAGCAG(-6)	TCCC	(-2)GACTGGGGGG(-2)	TC	AACTATGCTGAGCAGTTCTTCG(-1)
TCTCTGTGCCAGCA(-7)		(-3)ACTGGGGGGG(-1)		CTATGCTGAGCAGTTCTTCG(-3)

$RAG2^{FS/FS}$

TCTCTGTGCCAGCAGCTAAGA	AG	GGGACTGGGGG(-3)	A	AACTATGCTGAGCAGTTCTTCG(-1)
TCTCTGTGCCAGCAGCTAAG(-1)		GGGG	TCTTC	CTATGCTGAGCAGTTCTTCG(-3)
TCTCTGTGCCAGCAGCTAAG(-1)	T**C**	GGGACAGGGG(-2)	T	TGAGCAGTTCTTCG(-9)
TCTCTGTGCCAGCAGCTA(-3)		(-4)CAGGGGG(-1)		ACTATGCTGAGCAGTTCTTCG(-2)
TCTCTGTGCCAGCAGCTA(-3)	TT	(-5)TGGGGGGG(-1)	GG	TAACTATGCTGAGCAGTTCTTCG
TCTCTGTGCCAGCAGCTA(-3)	T			ACTATGCTGAGCAGTTCTTCG(-2)
TCTCTGTGCCAGCAGCTA(-3)	TCT	GGGACT(-8)	A	AACTATGCTGAGCAGTTCTTCG(-1)
TCTCTGTGCCAGCAGCT(-4)	T	(-1)GGACAG(-5)		TAACTATGCTGAGCAGTTCTTCG
TCTCTGTGCCAGCAGCT(-4)	T	GGGACAGG(-4)		AACTATGCTGAGCAGTTCTTCG(-1)
TCTCTGTGCCAGCAGCT(-4)		GGGACA(-6)	A	AACTATGCTGAGCAGTTCTTCG(-1)
TCTCTGTGCCAGCAGCT(-4)	CCTTC	(-1)GGACTGG(-6)	**A**	TAACTATGCTGAGCAGTTCTTCG
TCTCTGTGCCAGCAGCT(-4)	C**CCC**	GGGACAGG(-4)		ATGCTGAGCAGTTCTTCG(-5)
TCTCTGTGCCAGCAGCT(-4)	CTCGT	(-4)CAGGGGG(-1)	**A**	TAACTATGCTGAGCAGTTCTTCG
TCTCTGTGCCAGCAGCT(-4)	T	(-5)AGGGGGC	TCG	ATGCTGAGCAGTTCTTCG(-5)
TCTCTGTGCCAGCAGCT(-4)	T	(-5)TGGGGGG(-2)	CCGGGA	ATGCTGAGCAGTTCTTCG(-5)
TCTCTGTGCCAGCAGCT(-4)		(-7)GGGGGGC	CG	ACTATGCTGAGCAGTTCTTCG(-2)
TCTCTGTGCCAGCAGCT(-4)	C	(-4)CAGGGGGC		CTATGCTGAGCAGTTCTTCG(-3)
TCTCTGTGCCAGCAGC(-5)	CC**CCC**	GGGACA(-6)	AT	ACTATGCTGAGCAGTTCTTCG(-2)
TCTCTGTGCCAGCAGC(-5)	**CC**	GGGACAGGGG(-4)		TATGCTGAGCAGTTCTTCG(-4)
TCTCTGTGCCAGCAGC(-5)	A**CC**	GGGACTGGGGGGG	T	CTATGCTGAGCAGTTCTTCG(-3)
TCTCTGTGCCAGCAGC(-5)	A	GGGACAG(-5)	C	AACTATGCTGAGCAGTTCTTCG(-1)
TCTCTGTGCCAGCAGC(-5)	AA**CC**	GGGACT(-8)	T	TAACTATGCTGAGCAGTTCTTCG
TCTCTGTGCCAGCAGC(-5)	CCC	(-2)GACTGGGGG(-3)	AT	CTATGCTGAGCAGTTCTTCG(-3)
TCTCTGTGCCAGCAGC(-5)	CCTC	(-6)GGGGGGG(-1)	A	AACTATGCTGAGCAGTTCTTCG(-1)
TCTCTGTGCCAGCAGC(-5)	CGTGAC	GGG		ATGCTGAGCAGTTCTTCG(-5)
TCTCTGTGCCAGCAGC(-5)	CCC	(-2)GACAGGG(-3)	**A**	TAACTATGCTGAGCAGTTCTTCG
TCTCTGTGCCAGCAGC(-5)	C**CCC**	GGGACT(-8)	TG	AACTATGCTGAGCAGTTCTTCG(-1)
TCTCTGTGCCAGCAG(-6)	TTT	GGGAC(-7)	CGAAA	TATGCTGAGCAGTTCTTCG(-4)
TCTCTGTGCCAGCAG(-6)	GT**CC**	GGGACAGGGGG	GG	ATGCTGAGCAGTTCTTCG(-5)
TCTCTGTGCCAGCAG(-6)	GAA	(-2)GACAGG(-4)	TT	ACTATGCTGAGCAGTTCTTCG(-2)
TCTCTGTGCCAGC(-8)	**CC**	GGGACTGGGG(-4)		CTATGCTGAGCAGTTCTTCG(-3)
TCTCTGTGCCAGC(-8)		(-5)TGGGGGG(-2)	T	CTATGCTGAGCAGTTCTTCG(-3)

Fig.S4

C

Vβ14		Dβ1		Jβ1.1
CCTCTGTGCCTGGAGTCT		GGGACAGGGGGC		CAAACACAGAAGTCTTCTTTGG

WT

CCTCTGTGCCTGGAGTCT	**AG**TG	CAGG		CAAACACAGAAGTCTTCTTTGG
CCTCTGTGCCTGGAGTCT	**AG**	GGGACAGGGGG	T	CAAACACAGAAGTCTTCTTTGG
CCTCTGTGCCTGGAGTCT	G	AGGG	**AAG**	CAAACACAGAAGTCTTCTTTGG
CCTCTGTGCCTGGAGTCT		GG	TTCCTTTAC	CAAACACAGAAGTCTTCTTTGG
CCTCTGTGCCTGGAGTCT	**ATT**	GGGG		AACACAGAAGTCTTCTTTGG(-2)
CCTCTGTGCCTGGAGTCT	**A**	GACA	ATA	AGAAGTCTTCTTTGG(-7)
CCTCTGTGCCTGGAGTCT	**AGA**	GC	T	GTCTTCTTTGG(-11)
CCTCTGTGCCTGGAGTC(-1)	CAGA**CC**	GGGACAG	C	AACACAGAAGTCTTCTTTGG(-2)
CCTCTGTGCCTGGAGT(-2)	TCA	GGGACAGGGGGC	**G**GAGG	AGTCTTCTTTGG(-10)
CCTCTGTGCCTGGA(-4)		ACAGGGG		AAACACAGAAGTCTTCTTTGG(-1)
CCTCTGTGCCT(-7)	**C**	GGGACAGGG	AAGG	ACAGAAGTCTTCTTTGG(-5)

RAG2$^{FS/FS}$

CCTCTGTGCCTGGAGTCT	**AG**GC	AC	TA	CAAACACAGAAGTCTTCTTTGG
CCTCTGTGCCTGGAGTCT	**AG**	AGGGG	**TC**	CAAACACAGAAGTCTTCTTTGG
CCTCTGTGCCTGGAGTCT	G	AGGG	**AAG**	CAAACACAGAAGTCTTCTTTGG
CCTCTGTGCCTGGAGTCT	**AAT**	AGGG		AAACACAGAAGTCTTCTTTGG(-1)
CCTCTGTGCCTGGAGTCT		AGGGGG	G	AACACAGAAGTCTTCTTTGG(-2)
CCTCTGTGCCTGGAGTCT	**A**CC	GACAG	T	CAGAAGTCTTCTTTGG(-6)
CCTCTGTGCCTGGAGTCT	**A**	GACAGGG	AC	CAGAAGTCTTCTTTGG(-6)
CCTCTGTGCCTGGAGTCT	TTA	ACAGGGGGC	AA	AAGTCTTCTTTGG(-9)
CCTCTGTGCCTGGAGTC(-1)	GAAGGG	AGGGG	T	CACAGAAGTCTTCTTTGG(-4)
CCTCTGTGCCTGGAGTC(-1)		GACAG	ATGT	CAGAAGTCTTCTTTGG(-6)
CCTCTGTGCCTGGAGT(-2)	GT	AG	TAAC	CACAGAAGTCTTCTTTGG(-4)
CCTCTGTGCCTGGAG(-3)		CAGGGGG	**G**	CAAACACAGAAGTCTTCTTTGG
CCTCTGTGCCTGG(-5)	GAT	ACAGG	TTT	AGAAGTCTTCTTTGG(-7)
CCTCTGTGCC(-8)	CT	CAGGG	G**TG**	CAAACACAGAAGTCTTCTTTGG

D

Dβ2		
**tttttgtatcacgatgtaacattgtg**GGGACTGGGGGGGC		CTCCTATGAACAGTACTTCGGTCCCGG Jβ2.6
		AACCAAGACACCCAGTACTTTGGGCCA Jβ2.5
		AGTCAAAACACCTTGTACTTTGGTGCG Jβ2.4

WT

**tttttgtatcacgatgtaacattgtg**GGGACTGGGGGGGC	**GC**TTGGGGC	CCAAGACACCCAGTACTTTGGGCCA(-2)
**tttttgtatcacgatgtaacattgtg**GGGACTGGGGGGGC	**G**ATGGG	CAAGACACCCAGTACTTTGGGCCA(-3)
**tttttgtatcacgatgtaacattgtg**GGGACTGGGGGGGC	**GC**TT	AGACACCCAGTACTTTGGGCCA(-5)
**tttttgtatcacgatgtaacattgtg**GGGACTGGGGGGGC	**GC**T	TGAACAGTACTTCGGTCCCGG(-6)
**tttttgtatcacgatgtaacattgtg**GGGACTGGGGGGGC	CC	CACCCAGTACTTTGGGCCA(-8)
**tttttgtatcacgatgtaacattgtg**GGGACTGGGGGGG(-1)	T	TCCCGG(-21)
**tttttgtatcacgatgtaacattgtg**GGGACTGGGGGGG(-1)	G	CTATGAACAGTACTTCGGTCCCGG(-3)
**tttttgtatcacgatgtaacattgtg**GGGACTGGGGGGG(-1)	GT	TATGAACAGTACTTCGGTCCCGG(-4)
**tttttgtatcacgatgtaacattgtg**GGGACTGGGGGGG(-1)	GG	CCAAGACACCCAGTACTTTGGGCCA(-2)
**tttttgtatcacgatgtaacattgtg**GGGACTGGGGGGG(-1)		AAGACACCCAGTACTTTGGGCCA(-4)
**tttttgtatcacgatgtaacattgtg**GGGACTGGGGGG(-2)	T	TGAACAGTACTTCGGTCCCGG(-6)
**tttttgtatcacgatgtaacattgtg**GGGACTGGGGGG(-2)	AGGCG	GACACCCAGTACTTTGGGCCA(-6)
**tttttgtatcacgatgtaacattgtg**GGGACTGGGGG(-3)		CCAAGACACCCAGTACTTTGGGCCA(-2)
**tttttgtatcacgatgtaacattgtg**GGGACTGGG(-5)	CC**TT**	AACCAAGACACCCAGTACTTTGGGCCA
**tttttgtatcacgatgtaacattgtg**GGGACTGGG(-5)	**TT**	AACCAAGACACCCAGTACTTTGGGCCA

RAG2$^{FS/FS}$

**tttttgtatcacgatgtaacattgtg**GGGACTGGGGGGGC	ACGAG	CCAAGACACCCAGTACTTTGGGCCA(-2)
**tttttgtatcacgatgtaacattgtg**GGGACTGGGGGGGC	**GC**AGG	CCAAGACACCCAGTACTTTGGGCCA(-2)
**tttttgtatcacgatgtaacattgtg**GGGACTGGGGGGGC	CGC	CCCAAGACACCCAGTACTTTGGGCCA(-2)
**tttttgtatcacgatgtaacattgtg**GGGACTGGGGGGGC		TCCTATGAACAGTACTTCGGTCCCGG(-1)
**tttttgtatcacgatgtaacattgtg**GGGACTGGGGGGGC	CT**T**	AGTCAAAACACCTTGTACTTTGGTGCG
**tttttgtatcacgatgtaacattgtg**GGGACTGGGGGGGC		CCAAGACACCCAGTACTTTGGGCCA(-2)
**tttttgtatcacgatgtaacattgtg**GGGACTGGGGGGGC		CCAAGACACCCAGTACTTTGGGCCA(-2)
**tttttgtatcacgatgtaacattgtg**GGGACTGGGGGGGC		GACACCCAGTACTTTGGGCCA(-6)
**tttttgtatcacgatgtaacattgtg**GGGACTGGGGGGG(-1)	AA**ACT**	AGTCAAAACACCTTGTACTTTGGTGCG
**tttttgtatcacgatgtaacattgtg**GGGACTGGGGGGG(-1)	G	ACACCTTGTACTTTGGTGCG(-7)
**tttttgtatcacgatgtaacattgtg**GGGACTGGGGGGG(-1)		AGACACCCAGTACTTTGGGCCA(-5)
**tttttgtatcacgatgtaacattgtg**GGGACTGGGGGG(-2)	TG	AACACCTTGTACTTTGGTGCG(-6)
**tttttgtatcacgatgtaacattgtg**GGGACTGGGGG(-3)	AT	TATGAACTTCGGTCCCGG(-4)
**tttttgtatcacgatgtaacattgtg**GGGACTGGGGG(-3)		TTTGGGCCA(-18)
**tttttgtatcacgatgtaacattgtg**GGGACTGGGG(-4)		AACCAAGACACCCAGTACTTTGGGCCA
**tttttgtatcacgatgtaacattgtg**GGGACTGGG(-5)	AG	GACACCCAGTACTTTGGGCCA(-6)
**tttttgtatcacgatgtaacattgtg**GGGACTGG(-6)	TGGATG	AAAACACCTTGTACTTTGGTGCG(-4)

Fig.S4

E

Vh7183			Jh4
CTTGTATTACTGTGCAAGACA	TTTATTACTACGGTAGTAGCTAC		ATTACTATGCTATGGACTACTGG
AGCCATGTATTACTGTGCAAGAGA	TCTATGATGGTTACTAC		
	TCTACTATGATTACGAC		
	CCTACTATAGTAACTAC		
	TCTACTATGGTTACGAC		
	TCTACTATGGTAACTAC		
	CTAACTGGGAC		

WT

CTTGTATTACTGTGCAAGACA		GGGT		TATGCTATGGACTACTGG(-5)
CTTGTATTACTGTGCAAGACA	GGGGGAT	CTACTATGATTAC	GTTCCCCGC	ATGGACTACTGG(-11)
CATGTATTACTGTGCAAGAGA	**T**GG	TTACTACG	AGAATGCTGA	GGACTACTGG(-13)
CTTGTATTACTGTGCAAGAC(-1)		TATGATTACGAC	GGGG	CTATGCTATGGACTACTGG(-4)
CTTGTATTACTGTGCAAGAC(-1)	GAGGAC	GGTAA	GGGGGTTTCAT	CTATGCTATGGACTACTGG(-4)
CATGTATTACTGTGCAAGAG(-1)	TTCCCTGA	TCTACTATGATTA	GGGG	CTATGCTATGGACTACTGG(-4)
CATGTATTACTGTGCAAGAG(-1)	G	ACTGGGAC	GTC	ACTATGCTATGGACTACTGG(-3)
CTTGTATTACTGTGCAAGA(-2)		ATTACTACGGTAGTAGC	G	ATGCTATGGACTACTGG(-6)
CTTGTATTACTGTGCAAG(-3)	G	CTACGGTAGT	CCC	ATTACTATGCTATGGACTACTGG
CTTGTATTACTGTGCAAG(-3)	GTGGG	ATGGTTAC	TC	TTACTATGCTATGGACTACTGG(-1)
CTTGTATTACTGTGCAAG(-3)	GCCCTC	TCTACT	CG	ACTATGCTATGGACTACTGG(-3)
CTTGTATTACTGTGCAAG(-3)	G	TACTATGG	GAGAAAGAGGGT	CTATGCTATGGACTACTGG(-4)
CTTGTATTACTGTGCAAG(-3)		CGTCAAGG		ATGCTATGGACTACTGG(-6)
CATGTATTACTGTGCAAG(-3)	GTCA**A**	TCTACTATGGTAACTAC	**G**GG	GCTATGGACTACTGG(-8)
CTTGTATTACTGTGCA(-5)	GT	TAACTGGGAC	TGGGA	GCTATGGACTACTGG(-8)
CTTGTATTACTGTGC(-6)	CCGCC	TGATGGT	CT	CTATGCTATGGACTACTGG(-4)
CTTGTATTACTGTGC(-6)	CTTGAACGA	TTTATTACTACGGTAGTAGCTAC	CGTGGA	TGGACTACTGG(-12)

RAG2$^{FS/FS}$

CTTGTATTACTGTGCAAGACA	CG	ATGATTAC	CCTT	ATTACTATGCTATGGACTACTGG
CATGTATTACTGTGCAAGAGA	**T**CGGG	GGGAC	CGC**T**	ATTACTATGCTATGGACTACTGG
CTTGTATTACTGTGCAAGACA	**TG**G	TTTAT		ATTACTATGCTATGGACTACTGG
CTTGTATTACTGTGCAAGACA		TGAT	ACGTCCTT	ATTACTATGCTATGGACTACTGG
CTTGTATTACTGTGCAAGACA	**TG**	ACTATAGTAACTAC	TT	TTACTATGCTATGGACTACTGG(-1)
CTTGTATTACTGTGCAAGACA		TGATTACGAC	G	TACTATGCTATGGACTACTGG(-2)
CATGTATTACTGTGCAAGAGA		TAAC	CCGG	ACTATGCTATGGACTACTGG(-3)
CTTGTATTACTGTGCAAGACA	**T**AG	TTATTACTACGGTAGTAG	AA	ACTATGCTATGGACTACTGG(-3)
CTTGTATTACTGTGCAAGACA		TACTACGG	**G**	CTATGCTATGGACTACTGG(-4)
CATGTATTACTGTGCAAGAGA		TTACTA	TAGTG	ATGCTATGGACTACTGG(-6)
CTTGTATTACTGTGCAAGACA		CTATAGTAACTAC	GAAGTTAC	ATGCTATGGACTACTGG(-6)
CATGTATTACTGTGCAAGAGA		GGG	T	GCTATGGACTACTGG(-8)
CATGTATTACTGTGCAAGAG(-1)	GGG**AGG**	CCTACTATAGTAACT	TTATCCTTC	ATTACTATGCTATGGACTACTGG
CTTGTATTACTGTGCAAGAC(-1)		G		GGACTACTGG(-13)
CTTGTATTACTGTGCAAGA(-2)		CATGATACTAAGG		ACTATGCTATGGACTACTGG(-3)
CTTGTATTACTGTGCAAGA(-2)	GTCGGCT	TACTATATAGTAACTA	TTTAAGG	CTATGCTATGGACTACTGG(-4)
CTTGTATTACTGTGCAAGA(-2)	C	TACTACGG	GG	TGCTATGGACTACTGG(-7)
CTTGTATTACTGTGCAAGA(-2)		GGGAAGGAG		GCTATGGACTACTGG(-8)
CATGTATTACTGTGCAAG(-3)	G	TCTACTATGG	**CC**CT	TATGGACTACTGG(-10)
AGCCATGTATTACTGTGC(-6)	CCT	CTACTATAGTAACTAC	GG	TGCTATGGACTACTGG(-7)
CATGTATTACTGT(-8)	ACGAGAGATGGGGG	CCTACTATA	CG	ATGCTATGGACTACTGG(-6)

F

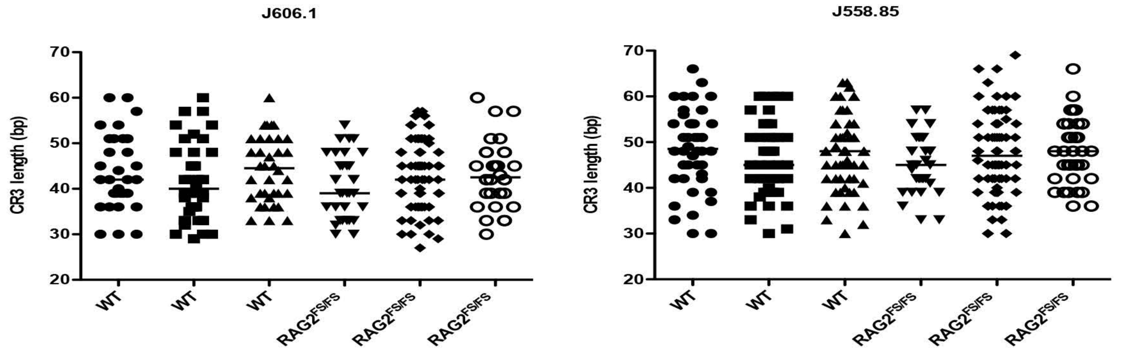

Fig.S4

A

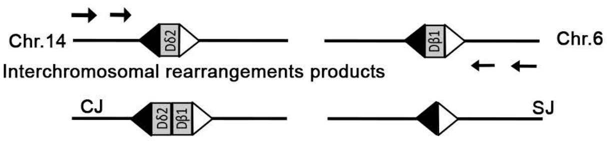

B

Dδ2		Dβ1
GGTTTTTGCAAAGCTCTGTAGCACCGTG		CACGGTGATTCAATTCTATGGGAAGCCTTTACAAAAACC
GGTTTTTGCAAAGCTCTGTAGCACCGTG		CACGGTGATTCAATTCTATGGGAAGCCTTTACAAAAACC
GGTTTTTGCAAAGCTCTGTAGCACCGTG	TCC	CACGGTGATTCAATTCTATGGGAAGCCTTTACAAAAACC
GGTTTTTGCAAAGCTCTGTAGCACCG(-2)	CGTCC	CACGGTGATTCAATTCTATGGGAAGCCTTTACAAAAACC
GGTTTTTGCAAAGCTCTGTAGCACCG(-2)	CG	CACGGTGATTCAATTCTATGGGAAGCCTTTACAAAAACC
GGTTTTTGCAAAG(-15)	G	GATTCAATTCTATGGGAAGCCTTTACAAAAACC(-6)
GGTTTTTGCAA(-17)	TAGG	GATTCAATTCTATGGGAAGCCTTTACAAAAACC(-6)
GG***TT***(-24)		CTGT(-42)
CCCA(-33)		CAATTCTATGGGAAGCCTTTACAAAAACC(-10)
AAAG(-37)	GCTG	TGGGAAGCCTTTACAAAAACC(-18)
attccaaggttt		GGAAGCCTTTACAAAAACC(-19)

Fig. S5

A

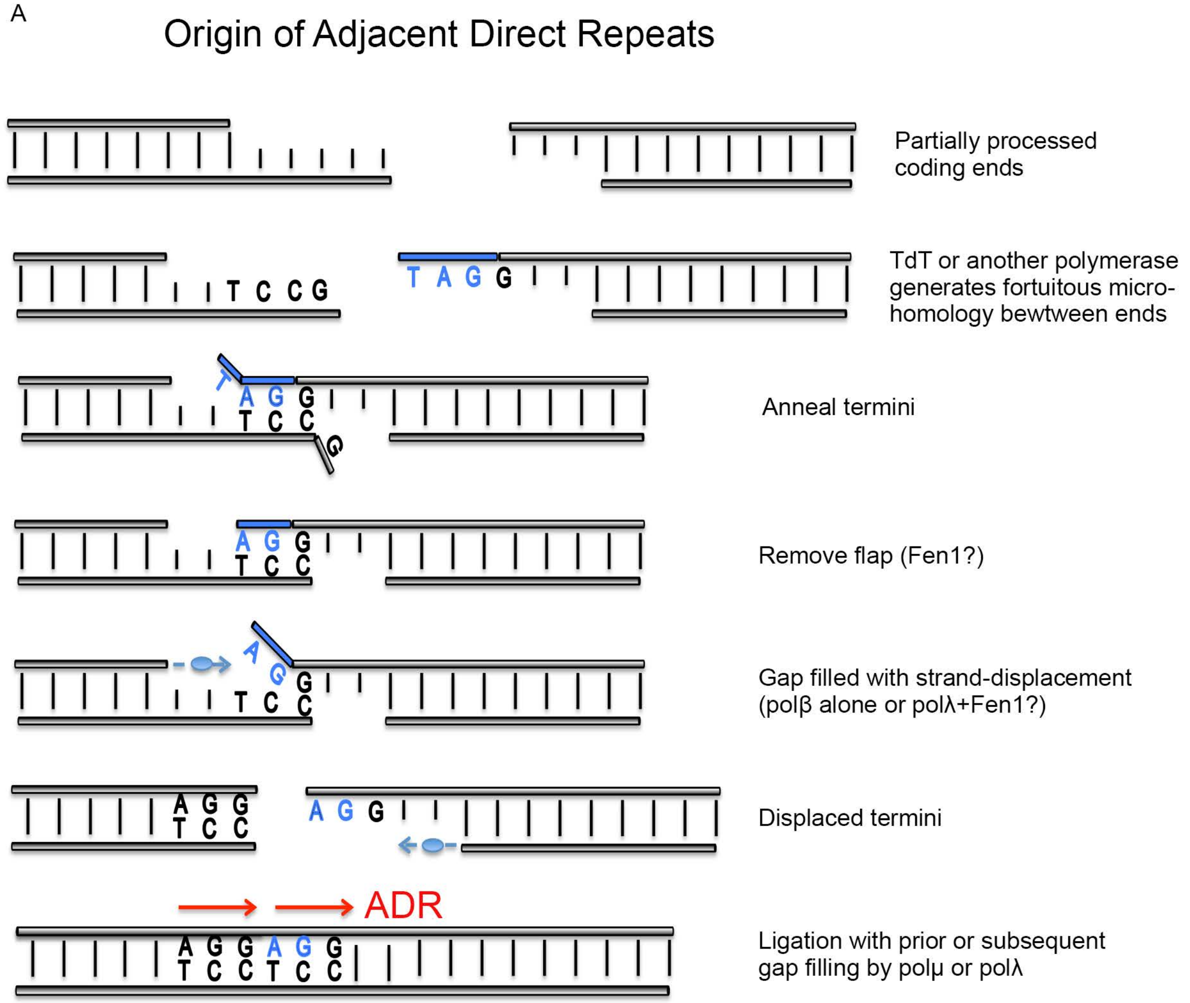

B

	Genotype	Vβ14-Jβ1.1	Vβ10-Jβ2.1	Vβ6/7/8-Jβ2	Dβ2-Jβ2.6
Talukder SR et al (25)	WT	3/40	1/30	nd	nd
Zhang L et al (Supp Info, 23)	WT	3/47	nd	nd	nd
Gigi V et al (This study)	WT	1/11	0/16	0/23	0/15
Gigi V et al (This study)	RAG2$^{FS/FS}$	1/14	0/32	2/31	2/17
Gigi V et al (This study)	RAG2$^{FS/FS}$; Ku80$^{-/-}$	5/22	1/12	nd	nd

Fig.S6

A

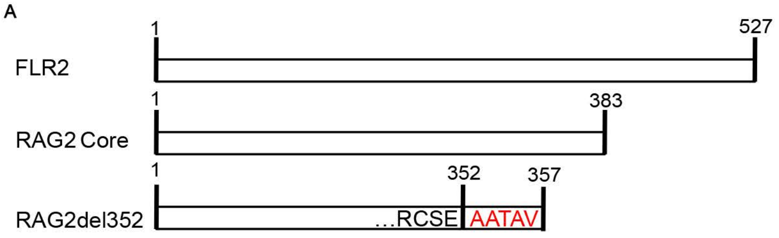

B

	**Vβ14-Dβ1 inversion**	**Vδ5-dδ2 inversion**	**Vβ8.3-Dβ1.1 Deletion**
Precise joints	60/85 (70%)	5/13 (38%)	7/30 (23%)
imprecise joints	25/85 (30%)	8/13 (58%)	23/30 (77%)
N addition	20/25 (80%)	6/8 (75%)	16/23 (69%)
Deletion	5/25 (20%)	8/8 (100%)	21/23 (91%)
%Deletions >5bp	4/8 (50%)	6/12 (50%)	20/22 (91%)
Miscleavage	3/25 (12%)	1/8 (12.5%)	19/23 (83%)
Microhomology≥2bp	0/25 (0%)	2/8 (25%)	0/23 (0%)

C

**Dδ2**		**Dβ1**
GGTTTTTGCAAAGCTCTGTAGCACCGTG		CACGGTGATTCAATTCTATGGGAAGCCTTTACAAAAACC
GGTTTTTGCAAAGCTCTGTAGCACCGTG		CACGGTGATTCAATTCTATGGGAAGCCTTTACAAAAACC
GGTTTTTGCAAAGCTCTGTAGCA(-5)	ACA	CACAGTCCTC(-59)
GGTTTTTGCAAAGCTCTGTAGC(-6)	CCATCGGC	CGGTGATTCAATTCTATGGGAAGCCTTTACAAAAACC(-2)
GGTTTTTGCAAAGCTCTGTAG(-7)	CCTGC	CGGTGATTCAATTCTATGGGAAGCCTTTACAAAAACC(-2)
GGTTTTTGCAAGCTCTGTA(-8)		cgccatcngccc
GGTTT(-23)		ctgacattaggt
tgccgattattc		CAATTCTATGGGAAGCCTTTACAAAAACC(-10)

D

**Vβ14**	**P/N nt**	**Dβ1**	**P/N nt**	**Jβ1.1**	**In frame**
GGCTTCTACCTCTGTGCCTGGAGTCT		GGGACAGGGGGC		CAAACACAGAAGTCTTCTTTGG	
GGCTTCTACCTCTGTGCCTGGAGTCT	**A**	ACAGGGGGC	C	CAGAAGTCTTCTTTGG(-6)	+
GGCTTCTACCTCTGTGCCTGGAGTCT		ACAGGGG	AGGG	AACACAGAAGTCTTCTTTGG(-2)	-
GGCTTCTACCTCTGTGCCTGGAGTCT	**AG**TC	CAGGGGG		CAAACACAGAAGTCTTCTTTGG	+
GGCTTCTACCTCTGTGCCTGGAGTCT	**A**	GGACAG		AACACAGAAGTCTTCTTTGG(-2)	+
GGCTTCTACCTCTGTGCCTGGAG(-3)	GATCCT**CC**	GGGACAG		ACACAGAAGTCTTCTTTGG(-4)	-
GGCTTCTACCTCTGTGCCT(-4)		***GGA***CAGGGGGC	T	CAAACACAGAAGTCTTCTTTGG	+
GGCTTCTACCTCTGTGCCTGGA(-4)	C**CCC**	GGGACA	A	CAAACACAGAAGTCTTCTTTGG	-
GGCTTCTACCTCTG(-12)				ACAGAAGTCTTCTTTGG(-5)	-

**Vβ10**	**P/N nt**	**Dβ1/2**	**P/N nt**	**Jβ2.1**	**In frame**
TGTGTATCTCTGTGCCAGCAGCTAAGA		GGGACAGGGGGC<br>GGGACTGGGGGGGC		TAACTATGCTGAGCAGTTCTTCG	
TGCTGTGTATCTCTGTGCCAGCAGC(-5)	CCT	GGGACTGGGG		TAACTATGCTGAGCAGTTCTTCG	-
TGCTGTGTATCTCTGTGCCAGCAG(-6)	GT**CC**	GGGACAGGGGG	GG	ATGCTGAGCAGTTCTTCG(-5)	+
TGCTGTGTATCTCTGTGCCAGCAG(-6)	GCC	GACTGGGGGGG	GG	AACTATGCTGAGCAGTTCTTCG(-1)	+
TGCTGTGTATCTCTGTGCCAGCAG(-6)		ACTGGGGGGG		CTATGCTGAGCAGTTCTTCG(-3)	-
TGCTGTGTATCTCTGTGCCAGCAG(-6)	AG	GGGGGGGC	**GC**	CTATGCTGAGCAGTTCTTCG(-3)	+

**Vα8**	**P/N nt**	**Jα58**	**In frame**
CCCTGTACTACTGTGCTCTGAGTGA<br>CTGTACTACTGTGCTTTGAGTGA		CAGCAAGGCACTGGGTCTAAGCT	
CTGTACTACTGTGCTCTGAGTGA	**TCA**GGGT	GGCACTGGGTCTAAGCT(-6)	+
CTGTACTACTGTGCTTTGAGTGA	**T**GCCTTC**G**	CAGCAAGGCACTGGGTCTAAGCT	-
CTGTACTACTGTGCTTTGAGTGA	GGG	AAGGCACTGGGTCTAAGCT(-4)	-
CTGTACTACTGTGCTCTGAGTG(-1)	TCCTC**G**	CAGCAAGGCACTGGGTCTAAGCT	-
CTGTACTACTGTGCTCTGAGTG(-1)	TAC**G**	CAGCAAGGCACTGGGTCTAAGCT	-
CTGTACTACTGTGCTTTGAGTG(-1)	CGA**G**	CAGCAAGGCACTGGGTCTAAGCT	-
CTGTACTACTGTGCTTTGAGT(-2)		AGCAAGGCACTGGGTCTAAGCT(-1)	-
CTGTACTACTGTGCTCTGAG(-3)	CGTTA	CAGCAAGGCACTGGGTCTAAGCT	-
CTGTACTTCTGTGCTTTG(-5)	GATACT	CAGCAAGGCACTGGGTCTAAGCT	+

Fig. S7

A

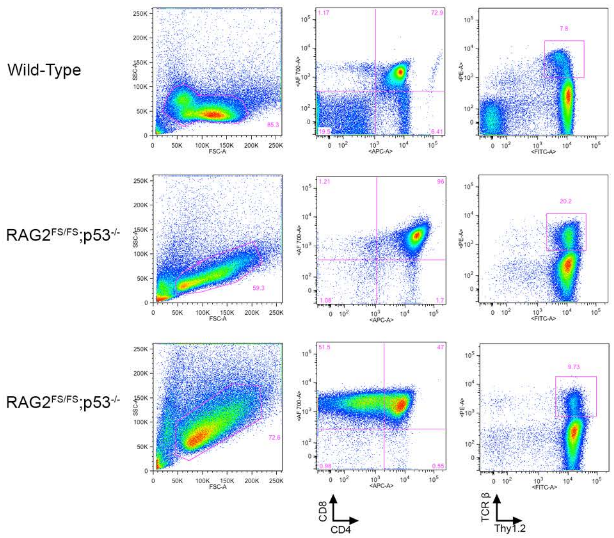

B

	End1 (23RSS)	P/N nt	End2 (12RSS)		
1	GCAAATACCTTGTGAAAGCC	CCAACCCCT	ATCCAGACTGCACAGTAGTA	TCRg	Del
2	CTTGTGAAAACCTGAGCTAT	CGT**A**	TGTACTGTTCTCTTGAGAATCG	TCRg	Del
3	GCGGTGATGGGGACAGGGGG	AAG	AACACAGAAGTCTTCTTTGGTA	TCRb	Del
4	GTGCCTTGGCCCCAGTAGTC	CCT	TCCCAGTTAGCACTGTGGTGCT	IgA	Del
5	AACATTGTGGGGACAGGGGG	AAG	AACACAGAAGTCTTCTTTGGTA	Db1-Jb1.1	Del
6	ACATTGTGGGGACAGGGGGC	**G**A	GAACAGTACTTCGGTCCCGGCA	Db1-Jb2.6	Del
7	TACCACTGCCTTCGGGGAGA	CCTT	TTCCAATACCAACAAAGTCGTC	TCRa	Del

Fig. S8
